# A Modified Slime Mould Algorithm for Global Optimization

**DOI:** 10.1155/2021/2298215

**Published:** 2021-11-24

**Authors:** An-Di Tang, Shang-Qin Tang, Tong Han, Huan Zhou, Lei Xie

**Affiliations:** Aeronautics Engineering College, Air Force Engineering University, Xi'an 710038, China

## Abstract

Slime mould algorithm (SMA) is a population-based metaheuristic algorithm inspired by the phenomenon of slime mould oscillation. The SMA is competitive compared to other algorithms but still suffers from the disadvantages of unbalanced exploitation and exploration and is easy to fall into local optima. To address these shortcomings, an improved variant of SMA named MSMA is proposed in this paper. Firstly, a chaotic opposition-based learning strategy is used to enhance population diversity. Secondly, two adaptive parameter control strategies are proposed to balance exploitation and exploration. Finally, a spiral search strategy is used to help SMA get rid of local optimum. The superiority of MSMA is verified in 13 multidimensional test functions and 10 fixed-dimensional test functions. In addition, two engineering optimization problems are used to verify the potential of MSMA to solve real-world optimization problems. The simulation results show that the proposed MSMA outperforms other comparative algorithms in terms of convergence accuracy, convergence speed, and stability.

## 1. Introduction

Solving optimization problems means finding the best value of the given variables which satisfies the maximum or minimum objective value without violating the constraints. With the continuous development of artificial intelligence technology, real-world optimization problems are becoming more and more complex. Traditional mathematical methods find it difficult to solve nonproductivity and noncontinuous problems effectively and are easily trapped in local optima [1, 2]. Metaheuristic optimization algorithms are able to obtain optimal or near-optimal solutions within a reasonable amount of time [3]. Thus they are widely used for solving optimization problems, such as mission planning [4–7], image segmentation [8–10], feature selection [11–13], and parameter optimization [14–18]. Metaheuristic algorithms find optimal solutions by modeling physical phenomena or biological activities in nature. These algorithms can be divided into three categories: evolutionary algorithms, physics-based algorithms, and swarm-based algorithms. Evolutionary algorithms, as the name implies, are a class of algorithms that simulate the laws of evolution in nature. Genetic algorithms [19] based on Darwin's theory of superiority and inferiority are one of the representatives. There are other algorithms such as differential evolution which mimic the crossover and variation mechanisms of genetics [20], biogeography-based optimization inspired by natural biogeography [21], and evolutionary programming [22] and evolutionary strategies [23]. Physics-based algorithms search for the optimum by simulating the laws or phenomena of physics in the universe. The simulated annealing, inspired by the phenomenon of metallurgical annealing, is the best-known physics-based algorithm. Apart from SA, other physics-based algorithms have been proposed, such as gravity search algorithm [24], sine cosine algorithm [25], black hole algorithm [26], nuclear reaction optimizer [27], and Henry gas solubility optimization [28]. Swarm-based algorithms are inspired from the social group behavior of animals or humans. Particle swarm optimization [29] and ant colony optimization [30], which simulate the foraging behavior of birds and ants, are two of the most common swarm-based algorithms. In addition to those, the researchers have proposed new swarm-based algorithms. The grey wolf optimizer [31] simulates the collaborative foraging of grey wolves. The salp swarm algorithm [32] is inspired by the foraging and following of the salps. Monarch butterfly optimization [33] is inspired by the migratory activities of monarch butterfly populations. The naked mole-rat algorithm [34] mimics the mating patterns of naked mole-rats. However, the no free lunch theory points out that no single algorithm can solve all optimization problems well [35]. This motivates us to continuously propose new algorithms and improve existing ones. Recently, inspired by the phenomenon of slime oscillations, Li proposed a new population-based algorithm called slime mould algorithm (SMA) [36]. Although SMA is competitive compared to other algorithms, there are some shortcomings in SMA. Due to the shortcoming of diminished population diversity in SMA, it easily falls into local optimum [37]. The selection of update strategies by SMA weakens the exploration ability [38, 39]. As the problem grows more complex, SMA converges slower in late iterations and has difficulty maintaining a balance between exploitation and exploration [40, 41]. To further enhance the performance of SMA and considering that the NFL encourages us to continuously improve these existing algorithms, a modified variant of SMA called MSMA is proposed in this paper. A chaotic opposition-based learning strategy is first used to improve population diversity. The search scope is expanded using the inverse solution of the imposed chaos operator. Second, two adaptive parameter control strategies are proposed to balance the relationship between exploitation and exploration better. Finally, a spiral search strategy is introduced to enhance the global exploration ability of the algorithm and avoid falling into local optimum. To verify the superiority of MSMA, 13 functions with variable dimensions and 10 functions with fixed dimensions were used for testing. The differences between the algorithms were also analyzed using the Wilcoxon test and the Friedman test. Moreover, two engineering optimization problems were used to verify the performance of MSMA further.

The remainder of this paper is organized as follows. A review of the basic SMA is provided in Section 2.  Section 3 provides a detailed description of the proposed MSMA. In Section 4, the effectiveness of the proposed improved strategy and the superiority of the improved algorithm are verified using classical test functions. Based on this, the MSMA is applied to solve the two engineering design problems in Section 4. The main reasons for the success of MSMA are discussed in Section 5. Finally, conclusions and future works are given in Section 6.

## 2. Slime Mould Algorithm

In this section, the basic procedure of SMA is described. SMA works by simulating the behavioral and morphological changes of slime mould during the foraging process. The mathematical model of the slime mould is as follows:

(1) $$\begin{array}{c} X_{t+1}=\left\{\begin{array}{cc} X_{t}^{\text{best}}+vb\cdot \left(W\cdot X_{t}^{A}-X_{t}^{B}\right), & \text{rand}<p,\\ vc\cdot X_{t}, & \text{rand}\geq p, \end{array}\right. \end{array}$$

where *t* denotes the number of current iterations. *X*_*t*_^best^ denotes the optimal individual. *X*_*t*_^*A*^ and *X*_*t*_^*B*^ are two individuals randomly selected from the population at iteration *t*. *vb* is a parameter in the range of −*a* to *a*. *vc* is a variable decreasing from 1 to 0. *W* denotes the weight of slime mould. *p* is a variable that is calculated by the following formula:

(2) $$\begin{array}{c} p=\tanh\left|S\left(i\right)-DF\right|,\;i=1,2,3,\ldots ,NP, \end{array}$$

where *S*(*i*) is the fitness of *X*. *NP* is the population number. *DF* is the best fitness so far.

The formula for *a* is as follows:

(3) $$\begin{array}{c} a=\arctan\;h\left(-\left(\frac{t}{t_{\max}}\right)+1\right), \end{array}$$

where *t*_max_ is the maximum number of iterations.

The formula of *W* is calculated as follows:

(4) $$\begin{array}{c} W\left(\text{smellindex}\left(i\right)\right)=\left\{\begin{array}{c} 1+\text{rand}\cdot \;\log\left(\frac{bF-S\left(i\right)}{bF-\omega F}\right),\;\text{condition,}\\ 1-\text{rand}\cdot \;\log\left(\frac{bF-S\left(i\right)}{bF-\omega F}\right),\;\text{others,} \end{array}\right. \end{array}$$

(5) $$\begin{array}{c} \text{smellindex}=\text{sort}\left(S\right), \end{array}$$

where condition denotes individuals ranking in the top half of fitness. *bF* and *ωF* denote the best fitness and worst fitness in the current population, respectively.

The mathematical formula for updating the position of the slime mould is as follows:

(6) $$\begin{array}{c} X_{t+1}=\left\{\begin{array}{cc} \text{rand}\cdot \left(ub-lb\right)+lb, & \text{rand}<z,\\ X_{t}^{\text{best}}+vb\cdot \left(W\cdot X_{t}^{A}-X_{t}^{B}\right), & \text{rand}<p,\\ vc\cdot X_{t}, & \text{rand}\geq p, \end{array}\right. \end{array}$$

where *ub* and *ub* are the upper and lower bounds of the search space, respectively.

## 3. Proposed MSMA

To overcome the shortcomings of the basic SMA, this paper proposes three improvement strategies to enhance its performance. A chaotic opposition-based learning strategy is used to enhance the population diversity, as well as balancing algorithm exploitation and exploration ability using self-adaptive strategy. A spiral search strategy is used to prevent the algorithm from falling into local optimum. The three improvement strategies are described in detail in the following.

### 3.1. Chaotic Opposition-Based Learning Strategy

Opposition-based learning (OBL) is a new technique that has emerged in recent years in computing, proposed by Tizhoosh [42]. It has been shown that the probability that the reverse solution gets closer to the global optimal solution is nearly 50% higher than that of the current original solution. OBL enhances population diversity mainly by generating the reverse position of each individual and evaluating the original and reverse individuals to retain the dominant individuals into the next generation. The OBL formula is as follows:

(7) $$\begin{array}{c} X_{i}^{o}=lb+ub-X_{i}^{t}, \end{array}$$

where *X*_*i*_^*o*^ is the reverse solution corresponding to *X*_*i*_^*t*^.

To further enhance the population diversity and overcome the deficiency that the reverse solution generated by the basic OBL is not necessarily better than the current solution, considering that chaotic mapping has the characteristics of randomness and ergodicity, it can help to generate new solutions and enhance the population diversity. Therefore, this paper combines chaotic mapping with OBL and proposes a chaotic opposition-based learning strategy. The specific mathematical model is described as follows:

(8) $$\begin{array}{c} X_{i}^{To}=lb+ub-\lambda_{i}\cdot X_{i}^{t}, \end{array}$$

where *X*_*i*_^*To*^ denotes the inverse solution corresponding to the *i*th individual in the population. *λ*_*i*_ is the corresponding chaotic mapping value.

### 3.2. Self-Adaptive Strategy

#### 3.2.1. New Nonlinear Decreasing Strategy

During the iterative optimization of SMA, the changes of parameter *a* have an important impact on the balance of exploitation and exploration. In SMA, *a* decreases rapidly in the early iterations and slows down in the later iterations. Smaller *a* in the early stage is not conducive to global exploration. Therefore, in order to further balance the exploitation and exploration and enhance the global exploration capability and the convergence capability of local exploitation, a new nonlinear decreasing strategy is proposed in this paper. The new definition of parameter *a* is shown as follows:

(9) $$\begin{array}{c} a=2\cdot {\left(\frac{1-t}{t_{\max}}\right)}^{\left(2\cdot t/t_{\max}\right)}. \end{array}$$

**Figure 1:**
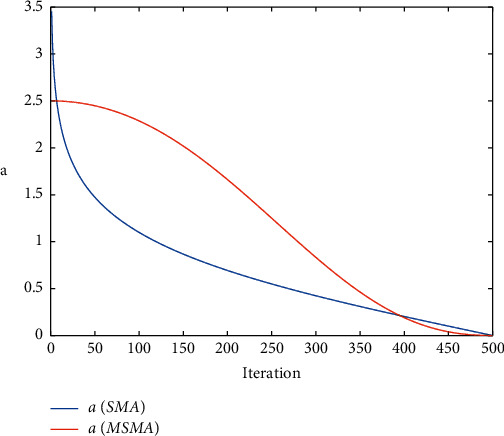
Comparison of parameter *a*.

To visually illustrate the effect of the new strategy, we compare it with the parameter change strategy in SMA, as shown in Figure 1. The new strategy proposed in this paper decreases slowly in the early stages, which increases the time for global exploration. In the late iteration, the reduction is also faster than the original strategy, which facilitates the SMA to accelerate the exploitation.

#### 3.2.2. Linear Decreasing Selection Range

For equation (1), the original SMA randomly selects two individuals from all populations. This is not conducive to the later convergence of the algorithm. In order to enhance the convergence of SMA, the selection range in equation (1) is reduced with increasing number of iterations. The selection range parameter SR is described as follows:

(10) $$\begin{array}{c} \text{SR}=\text{ceil}\left(\left(\frac{\left({\text{SR}}_{\min}-{\text{SR}}_{\max}\right)}{t_{\max}}\cdot t+{\text{SR}}_{\max}\right)\right), \end{array}$$

where SR_max_ and SR_min_ are the maximum and minimum selection ranges, respectively.

### 3.3. Spiral Search Strategy

**Figure 2:**
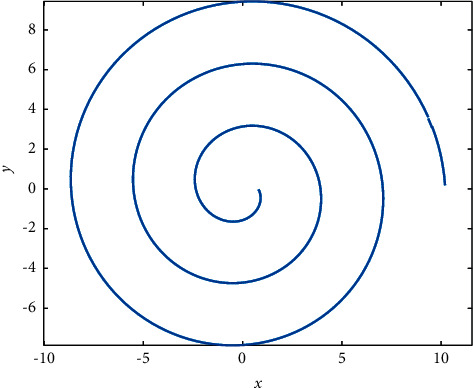
Spiral search schematic.

In order to better balance the exploitation and exploration of SMA, this paper introduces a spiral search strategy. The spiral search diagram is shown in Figure 2.

As can be seen from Figure 2, the spiral search strategy can expand the search scope and better improve the global exploration performance. The mathematical formula of the spiral search strategy is shown as follows:

(11) $$\begin{array}{c} X_{t+1}=X_{t}^{\text{best}}+e^{l}\cdot \;\cos\left(l\cdot 2\pi \right)\cdot \left(X_{t}^{\text{best}}-X_{t}\right),\\ l=1-2\left(\frac{t}{t_{\max}}\right). \end{array}$$

**Figure 3:**
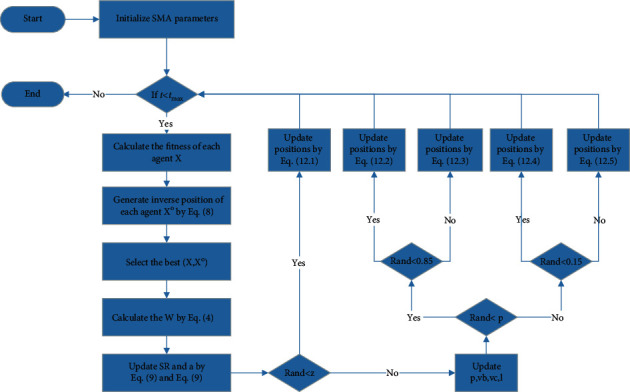
Flowchart of MSMA.

The spiral search strategy and the original strategy are chosen randomly according to the probability to update the population location. Thus, the modified position updating formula is as follows:

(12) $$\begin{array}{c} X_{t+1}=\left\{\begin{array}{cc} \text{rand}\cdot \left(ub-lb\right)+lb, & \text{rand}<z,\\ X_{t}^{\text{best}}+vb\cdot \left(W\cdot X_{t}^{A}-X_{t}^{B}\right), & \text{rand}<p\&\text{rand}<0.85,\\ X_{t}^{\text{best}}+e^{l}\cdot \;\cos\left(l\cdot 2\pi \right)\cdot \left(X_{t}^{\text{best}}-X_{t}\right), & \text{rand}<p\&\text{rand}\geq 0.85,\\ vc\cdot X_{t}, & \text{rand}\geq p\&\text{rand}<0.15,\\ X_{t}^{\text{best}}+e^{l}\cdot \;\cos\left(l\cdot 2\pi \right)\cdot \left(X_{t}^{\text{best}}-X_{t}\right), & \text{rand}\geq p\&\text{rand}\geq 0.15, \end{array}\right. \end{array}$$

and the pseudocode and flowchart of the MSMA are shown in Algorithm 1 and Figure 3.

### 3.4. Computational Complexity Analysis

MSMA is mainly composed of subsequent components: initialization, fitness assessment, reverse population fitness assessment, ranking, weight update, and position update, in which *Np* denotes the number of slime moulds, *D* denotes the function's dimension, and *T* denotes the maximum number of iterations. The computational complexity of initialization is*O*(*D*), fitness evaluation and inverse population fitness evaluation is *O*(*Np*+*Np*), the computational complexity of ranking is *O*(*NP* · log  *N*), the computational complexity of weight update is *O*(*Np* · *D*), and the complexity of position update is *O*(*Np* · *D*). Therefore, the total complexity of MSMA is *O*(*D*+*T* · *Np* · (2+log  *N*+*D*)).

## 4. Numerical Experiment and Analysis

In this section, various experiments are performed to verify the performance of MSMA. The experiments mainly include twenty-three classical test functions and two engineering design optimization problems.

### 4.1. Benchmark Test Functions and Parameter Settings

The 23 benchmark test functions include 7 unimodal, 6 multimodal, and 10 fixed-dimension multimodal functions. The unimodal functions have only one global optimal solution and are usually used to verify the exploitation capability of the algorithm. The multimodal functions have multiple locally optimal solutions and are therefore often used to examine the algorithm's exploration ability and its ability to escape from local optimums. These benchmark functions are listed in Table 1.

The experimental results of MSMA are compared with those of the other eight algorithms. The comparison algorithms are MPA [43], MFO [44], SSA [32], EO [45], MRFO [46], HHO [47], GSA, and GWO. To ensure fairness, all algorithms were run on a Windows 10 AMD R7 4800U 16 GB platform and code was programmed using MATLAB R2016b. In the experiment, the number of populations *NP* is 30 and the maximum number of iterations *t* is 500. The results of 50 independent runs of the experiment are recorded. The parameters of the comparison algorithms were set according to the original literature as shown in Table 2.

### 4.2. Chaotic Mapping Selection Test

The chaotic opposition-based learning strategy proposed in this paper combines chaotic mapping and opposition-based learning mechanisms. To verify which chaotic mapping is used, 10 chaotic mappings are combined with the opposition-based learning mechanism. The SMA using chaotic mapping with ID 1 is named SMA-C1. The rest of the SMA algorithms using chaotic mapping are named similarly. The details of the chaotic mappings are shown in Table 3.  Table 4 lists the results of each algorithm for solving the benchmark test functions.

As shown in Tables 4 and 5, SMA-C1, SMA-C2, SMA-C3, SMA-C4, SMA-C5, SMA-C6, SMA-C7, SMA-C8, SMA-C9, and SMA-C10 all show better results compared to SMA. This indicates that all 10 chaotic opposition-based learning strategies can improve SMA performance. SMA-C6 achieved the best results in solving the unimodal functions F1–F7, which indicates that the Pricewise map can better enhance the exploitation ability of SMA. When solving the multimodal functions F8–F13, SMA-C5 achieved satisfactory answers, which shows that the Logistic map can enhance the exploration ability of SMA. SMA-C4 achieves satisfactory solutions in the fixed-dimensional functions F14–F23, which indicates that Iterative map can enhance the local optimal avoidance ability of SMA. While SMA-C7 with Sine map is not the best performer in any of the three categories, it is ranked first in the overall ranking. This indicates that Sine map has the best effect in improving the comprehensive performance of SMA. In summary, in this paper, the Sine map with the first overall ranking is chosen to generate chaotic mapping values for chaotic opposition-based strategy.

### 4.3. Improvement Strategy Effectiveness Test

As seen in Section 3, three strategies are used in this paper to improve SMA performance. To evaluate the impact of each strategy on SMA, three SMA-derived algorithms (MSMA-1, MSMA-2, and MSMA-3) are developed according to Table 6. COBL represents chaotic opposition-based learning strategy, SA represents adaptive strategy, and SS represents spiral search strategy. Table 6 lists the results of each algorithm for solving the benchmark test functions.

**Figure 4:**
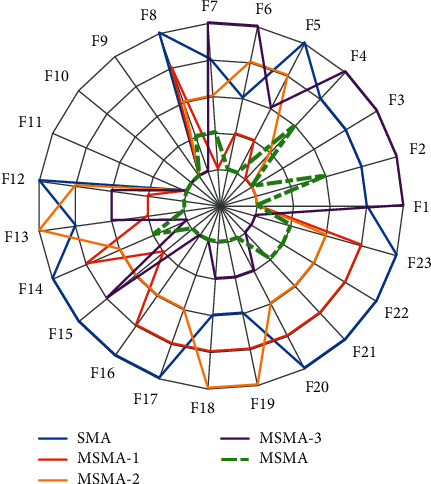
Ranking of improvement strategies.

As shown in Tables 7 and 8, MSMA with complete improvement strategies performs best overall. The three SMA-derived algorithms also ranked higher than SMA. The ranking from highest to lowest is as follows: MSMA-1, MSMA-2, and MSMA-3. It is shown that the three strategies have the largest to smallest impact on MSMA performance in the following order: COBL > SA > SS. Further analysis shows that MSMA-1 performs best in solving the unimodal functions F1–F7. This indicates that COBL can significantly improve the local search ability of SMA. MSMA-3 achieves satisfactory results on multimodal functions F8–F13 and F14–F23. This shows that SS can improve the global exploration capability of SMA, allowing the algorithm to get rid of local optimal solutions. MSMA-2 performs better in all three types of functions, which indicates that the adaptive strategy balances the exploitation and exploration capabilities of SMA. It is worth noting that MSMA-3 performs less well than SMA in the unimodal functions. This is due to the fact that the spiral search strategy expands the search of the space around the individual, resulting in a weakened exploitation capability. However, the combination of the three strategies allows the comprehensive performance of MSMA to be significantly improved, further illustrating the importance of balanced exploitation and exploration capabilities to enhance the performance of an algorithm. Finally, to more visually show the performance of each algorithm, a radar plot is drawn based on the ranking of each algorithm. As shown in Figure 4, the smaller the area enclosed by each curve, the better the performance. Obviously, MSMA has the smallest enclosed area, which means that MSMA has the best performance. On the contrary, SMA has the largest area.

### 4.4. Comparison and Analysis of Optimization Results

Tables 9 to Table 12 list the optimization results for F1–F13 of each algorithm for Dim = 30, 100, 500, 1000.  Table 13 then shows the results of the ten algorithms in fixed-dimensional functions F14–F23. From the optimization results, MSMA achieves better results in most of the test functions.

Specifically, for the unimodal functions F1–F7, MSMA achieved satisfactory results both in low dimensions and in high dimensions. MSMA can obtain the theoretical optimal solutions of F1 and F3 stably in different dimensions. In comparison, SMA failed to achieve the theoretical optimal value in all the test functions and performed weaker than MSMA. Comparing the test results of each dimension, we found that MSMA's performance has not dropped too much with increasing dimensions. This indicates that MSMA has excellent local exploitation capability. For the multimodal functions F8–F13, the MSMA steadily achieves the theoretical optimal values at F9–F11 with Dim = 30, 100, 500, 1000. When in low dimensions (Dim = 30, 100), MSMA performs best in solving F8. As the dimension increases, MSMA ranks second, only after SSA. MSMA has the best comprehensive performance in the multimodal functions, indicating that the improved strategy greatly enhances the global exploration capability of SMA.

Fixed-dimension functions are often used to test the ability of an algorithm to keep a balance between exploitation and exploration. The SMA performs best in six of the ten functions (F14, F16, F17, and F21–F23) when analyzing the mean and standard deviation. In addition, MSMA provides a better solution than SMA in all fixed-dimensional functions. Therefore, we can conclude that the MSMA proposed in this paper can well balance the exploitation and exploration capabilities with strong local optimal avoidance.

### 4.5. Convergence and Stability Analysis

**Figure 5:**
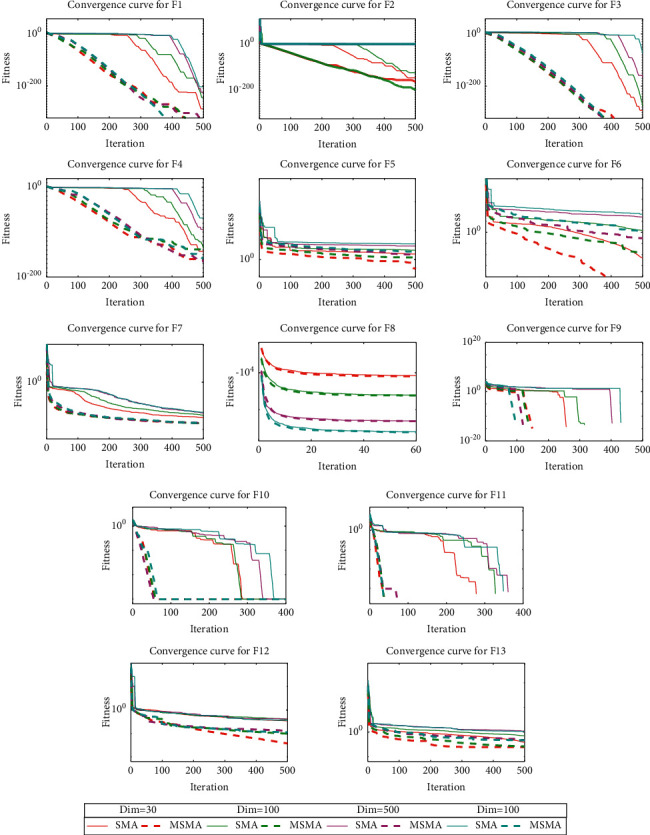
Convergence curves of SMA and MSMA on 13 test functions in 4 different dimensional cases.

In order to analyze the convergence performance of MSMA, convergence curves are plotted according to the results of different dimensions, as shown in Figure 5. We can learn that the convergence speed and convergence accuracy of MSMA are better than those of SMA in different dimensional cases. In addition, the convergence speed and convergence accuracy of MSMA do not decrease too much as the dimensionality increases. Therefore, the improvement strategy proposed in this paper can effectively improve the convergence speed of SMA and achieve better optimization results.

**Figure 6:**
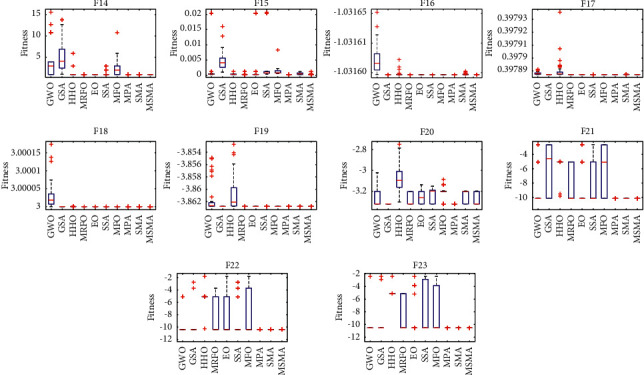
Boxplot analysis for fixed-dimensional functions.

To analyze the distribution properties of MSMA on a fixed-dimensional function, box plots were drawn. From Figure 6, it can be seen that the maximum, minimum, and median values of MSMA are almost the same in most of the test functions. Especially for F14 and F17, there are no outliers in MSMA. The above shows that MSMA is superior to the comparison algorithm in terms of stability.

### 4.6. Statistical Test

To statistically validate the differences between MSMA and the comparison algorithms, Wilcoxon's rank-sum test [48] and Friedman test [49] were used for testing.

Table 14 presents the statistical results with a significance level of 0.05. The symbols “+/=/−” indicate that MSMA is better than, similar to, or worse than the comparison algorithm. As shown in Table 14, MSMA outperforms other comparative algorithms in different cases and achieves results of 91/23/3, 96/18/3, 94/18/5, 93/19/5, and 66/15/9, confirming the significant superiority of MSMA in most cases compared to other algorithms.

Table 15 shows the statistics of F1–F13 in different dimensions and the fixed-dimensional functions F14–F23. The statistics show that MSMA ranks first in all cases. Therefore, it can be considered that MSMA has the best performance compared to other algorithms.

### 4.7. Engineering Design Problems

Engineering design optimization problems are often solved using metaheuristic algorithms. In this section, MSMA is used to solve two engineering design problems: the welded beam design problem and the tension/compression spring design problem. The results provided by MSMA are compared with those of other algorithms.

#### 4.7.1. Welded Beam Design Problem

**Figure 7:**
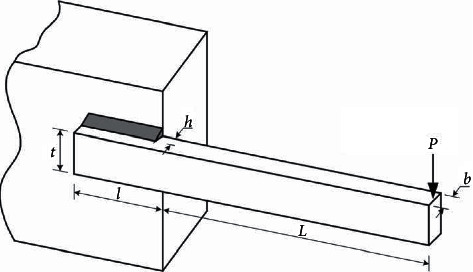
Schematic of welded beam design problem.

The welded beam design problem is a classical structural optimization problem, proposed by Coello [50]. As shown in Figure 7, the objective of this design problem is to minimize the manufacturing cost of the welded beam. The optimization variables include weld thickness *h* (*x*_1_), joint beam length *l* (*x*_2_), beam height *t* (*x*_3_), and beam thickness *b* (*x*_4_). The mathematical model of the welded beam design problem is as follows:

(13) $$\begin{array}{c} \min f\left(x_{1},x_{2},x_{3},x_{4}\right)=1.10471x_{1}^{2}x_{2}+0.04811x_{3}x_{4}\left(14.0+x_{2}\right). \end{array}$$

It is subject to

(14) $$\begin{array}{c} g_{1}\left(X\right)=\tau_{d}-\tau \left(X\right)\geq 0,\\ g_{2}\left(X\right)=\sigma_{d}-\sigma \left(X\right)\geq 0,\\ g_{3}\left(X\right)=x_{4}-x_{1}\geq 0,\\ g_{4}\left(X\right)=P_{c}\left(X\right)-P\geq 0,\\ g_{5}\left(X\right)=\delta_{d}-\delta \left(X\right)\geq 0, \end{array}$$

where

(15) $$\begin{array}{c} \tau \left(X\right)=\sqrt{{\left(\tau^{\prime }\left(X\right)\right)}^{2}+{\left(\tau^{\prime \prime }\left(X\right)\right)}^{2}+\frac{x_{2}\tau^{\prime }\left(X\right)\tau^{\prime \prime }\left(X\right)}{\sqrt{0.25\left(x_{2}^{2}+{\left(x_{1}+x_{3}\right)}^{2}\right)}}},\\ \sigma \left(X\right)=\frac{50400}{x_{3}^{2}x_{4}},\\ P_{c}\left(X\right)=64746.002\left(1-0.0282346x_{3}\right)x_{3}x_{4}^{3},\\ \delta \left(X\right)=\frac{2.1952}{x_{3}^{2}x_{4}},\\ \tau^{\prime }\left(X\right)=\frac{6000}{\sqrt{2}x_{1}x_{2}},\\ \tau^{\prime \prime }\left(X\right)=\frac{6000\left(14+0.5x_{2}\right)\sqrt{0.25\left(x_{2}^{2}+{\left(x_{1}+x_{3}\right)}^{2}\right)}}{2\left(0.707x_{1}x_{2}\left(x_{2}^{2}/12+0.25{\left(x_{1}+x_{3}\right)}^{2}\right)\right)}. \end{array}$$

The results of MSMA solving this problem are compared with those of other algorithms, as shown in Table 16. The results show that MSMA is the optimal algorithm for solving this problem, and the optimal solutions for each parameter are [0.205729, 3.470488, 9.036623, 0.205729], with the corresponding minimum cost of 1.724852.

#### 4.7.2. Tension/Compression Spring Design Problem

**Figure 8:**
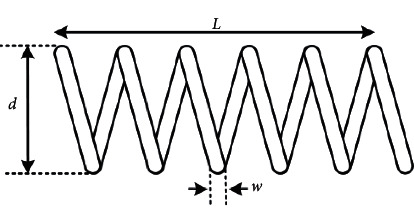
Schematic of tension/compression spring design problem.

The tension/compression spring design problem is a mechanical engineering design optimization problem. As shown in Figure 8, the objective of this problem is to reduce the weight of the spring. It includes three optimization objectives: wire diameter *w* (*x*_1_), average coil diameter *d* (*x*_2_), and the number of coils *L* (*x*_3_). The comparison results are shown in Table 17. The mathematical model of this problem is described below.


(16) $$\begin{array}{c} \min f\left(x_{1},x_{2},x_{3}\right)=\left(x_{3}+2\right)x_{1}^{2}x_{2},\\ \text{Subject to}\\ g_{1}\left(X\right)=1-\frac{x_{2}^{3}x_{3}}{71785x_{1}^{4}}\leq 0,\\ g_{2}\left(X\right)=\frac{x_{2}\left(4x_{2}-x_{1}\right)}{12566x_{1}^{3}\left(x_{2}-x_{1}\right)}+\frac{1}{5108x_{1}^{2}}-1\leq 0,\\ g_{3}\left(X\right)=1-\frac{140.45x_{1}}{x_{2}^{2}x_{3}}\leq 0,\\ g_{4}\left(X\right)=\frac{2\left(x_{1}+x_{2}\right)}{3}-1\leq 0,\\ \text{Variable range}:0.05\leq x_{1}\leq 2,0.25\leq x_{2}\leq 1.3,2.0\leq x_{3}\leq 15.0. \end{array}$$

The results showed that MSMA achieved the lowest cost of 0.012665 compared to GA3, CPSO, CDE, DDSCA, GSA, hHHO-SCA, AEO, and MVO. The corresponding values of the variables were [0.051747, 0.358090, 11.122192].

## 5. Discussion

In this section, the reasons for the superior performance of MSMA are discussed. The results in Table 5 demonstrate that the chaotic opposition-based learning strategy can enhance the performance of SMA. The different results of different chaotic mappings are caused by the different sequences generated by each chaotic mapping. The results reported in Table 8 demonstrate that all three improvement strategies proposed in this paper can improve the performance of the algorithm. MSMA-1 is competitive in unimodal functions. This is mainly due to the utilization of chaotic mapping for MSMA-1 to enhance the exploitation. MSMA-3 uses a spiral search strategy to improve the performance on the multimodal functions. It is due to the fact that the strategy expands the search of each individual for the space around itself and the population diversity is better. MSMA-2 maintains a balance of exploitation and exploration through adaptive strategies and thus ranks medium in both the multimodal and unimodal functions. The best performance of MSMA indicates that these three strategies complement each other and maintain a good balance between exploitation and exploration. This is also evidenced by the results of the Friedman test in Table 15.

## 6. Conclusions

In this paper, three improvement strategies are proposed in order to improve the performance of SMA. Firstly, a chaotic opposition-based learning strategy is used to enhance the population diversity. Secondly, two adaptive parameter control strategies are proposed to effectively balance the exploitation and exploration of SMA. Finally, a spiral search strategy is used to expand the SMA to search near individuals and avoid falling into local optimum. To evaluate the performance of the proposed MSMA, 23 classical test functions are used, including 13 multidimensional functions (Dim = 30, 100, 500, 1000) and 10 fixed-dimensional functions.

From the experimental results and the discussion just mentioned, the following conclusions can be drawn.

The sine mapping works best in combination with the opposition-based learning mechanism. Using chaotic opposition-based learning strategy can enhance the exploitation capability of MSMA.

Using a spiral search strategy can significantly enhance MSMA's exploration capabilities and avoid getting trapped in local optimum.

The two self-adaptive strategies maintain a good balance between exploitation and exploration.

Compared with the eight advanced algorithms, MSMA has better convergence accuracy, faster convergence speed, and more stable performance. MSMA has the potential to solve real-world optimization problems.

In future work, we will use MSMA to solve the multi-UAV path planning problem and the task assignment problem. Moreover, MSMA can be extended as a multiobjective optimization algorithm.

## Figures and Tables

**Algorithm 1:**
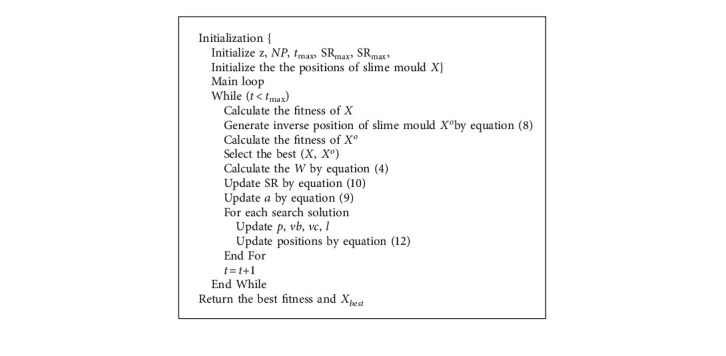
Pseudocode of the MSMA.

**Table 1:** The classic benchmark functions (M: multimodal, U: unimodal, S: separable, N: nonseparable, dim: dimension, range: limits of search space, optimum: global optimal value).

Test function	Name	Type	Dim	Range	Optimum
*f*_01_(*x*)=∑_*i*=1_^*D*^*x*_*i*_^2^	Sphere	US	30	[−100, 100]	0
*f*_02_(*x*)=∑_*i*=1_^*D*^|*x*_*i*_|+∏_*i*−1_^*D*^|*x*_*i*_|	Schwefel 2.22	UN	30	[−10, 10]	0
*f*_03_(*x*)=∑_*i*=1_^*D*^(∑_*j*−1_^*D*^*x*_*i*_)^2^	Schwefel 1.2	UN	30	[−100, 100]	0
*f*_04_(*x*)=max_*i*_{|*x*_*i*_|, 1 ≤ *i* ≤ *D*}	Schwefel 2.21	US	30	[−100, 100]	0
*f*_05_(*x*)=∑_*i*=1_^*D*^100(*x*_*i*+1_^2^ − *x*_*i*_^2^)^2^+(*x*_*i*_ − 1)^2^	Rosenbrock	UN	30	[−30, 30]	0
*f*_06_(*x*)=∑_*i*=1_^*D*^(⌊*x*_*i*_+0.5⌋)^2^	Step	US	30	[−100, 100]	0
*f*_07_(*x*)=∑_*i*=1_^*D*^*ix*_*i*_^4^+random[0,1)	Quartic	US	30	[−1.28, 1.28]	0

$f_{08}\left(x\right)=\sum_{i=1}^{D}-x_{i}\sin\left(\sqrt{\left|x_{i}\right|}\right)$	Schwefel 2.26	MS	30	[−500, 500]	−418.9829∗D
*f*_09_(*x*)=∑_*i*=1_^*D*^(*x*_*i*_^2^ − 10 cos(2*πx*_*i*_)+10)	Rastrigin	MS	30	[−5.12, 5.12]	0
$f_{10}\left(x\right)=20+e-20\;\exp\left(-0.2\sqrt{\left(1/D\right)\sum_{i=1}^{D}x_{i}^{2}}\right)-\exp\left(\left(1/D\right)\sum_{i=1}^{D}\cos\left(2\pi x_{i}\right)\right)$	Ackley	MS	30	[−32, 32]	8.8818*e* − 16
$f_{11}\left(x\right)=\left(1/4000\right)\sum_{i=1}^{D}\left(x_{i}^{2}\right)-\left(\prod_{i=1}^{D}\cos\left(x_{i}/\sqrt{i}\right)\right)+1$	Griewank	MN	30	[−600, 600]	0
$\begin{array}{c} f_{12}\left(x\right)=\left(\pi /D\right)\left\{10\;\sin^{2}\left(\pi y_{i}\right)+\sum_{i-1}^{D-1}{\left(y_{i}-1\right)}^{2}\left[1+10\;\sin^{2}\left(\pi y_{i+1}\right)\right]+\left(y_{D}-1\right)\right\}+\sum_{i-1}^{D}u\left(x_{i},10,100,4\right),\\ y_{i}=1+\left(x_{i}+1/4\right)u\left(x_{i},a,k,m\right)=\left\{\begin{array}{cc} k{\left(x_{i}-a\right)}^{m}, & x_{i}>a\\ 0, & -a<x_{i}<a\\ k{\left(-x_{i}-a\right)}^{m} & x_{i}<a \end{array}\right. \end{array}$	Penalized	MN	30	[−50, 50]	0
*f*_13_(*x*)=0.1{sin^2^(3*πx*_*i*_)+∑_*i*=1_^*D*^(*x*_*i*_ − 1)^2^[1+ sin^2^(3*πx*_*i*_)]+(*x*_*D*_ − 1)^2^[1+ sin^2^(2*πx*_*D*_)]}+∑_*i*−1_^*D*^*u*(*x*_*i*_, 5,100,4)	Penalized2	MN	30	[−50, 50]	0

*f*_14_(*x*)=((1/500)+∑_*j*=1_^25^(1/*j*+∑_*i*=1_^2^(*x*_*i*_ − *a*_*ij*_)^6^))^−1^	Foxholes	MS	2	[−65.53, 65.53]	0.998004
*f*_15_(*x*)=∑_*i*=1_^11^(*a*_*i*_ − (*x*_1_(*b*_*i*_^2^+*b*_*i*_*x*_2_)/*b*_*i*_^2^+*b*_*i*_*x*_3_+*x*_4_))^−1^	Kowalik	MS	4	[−5, 5]	0.0003075
*f*_16_(*x*)=4*x*_1_^2^ − 2.1*x*_1_^4^+1/3*x*_1_^6^+*x*_1_*x*_2_ − 4*x*_2_^2^+*x*_2_^4^	Six-Hump Camel Back	MN	2	[−5, 5]	−1.03163
*f*_17_(*x*)=(*x*_2_ − (5.1/4*π*^2^)*x*_1_^2^+(5/*π*)*x*_1_ − 6)^2^+10(1 − (1/8*π*))cos *x*_1_+10	Branin	MS	2	[−5, 10]×[0, 15]	0.398
*f*_18_(*x*)=[1+(*x*_1_+*x*_2_+1)^2^(19 − 14*x*_1_+3*x*_1_^2^ − 14*x*_2_+6*x*_1_*x*_2_+3*x*_2_^2^)] × [30+(2*x*_1_ − 3*x*_2_)^2^(18 − 32*x*_1_+12*x*_1_^2^+48*x*_2_ − 36*x*_1_*x*_2_+27*x*_2_^2^)]	Goldstein Price	MN	2	[−5, 5]	3
*f*_19_(*x*)=−∑_*i*=1_^4^(*c*_*i*_exp(−∑_*j*−1_^3^*a*_*ij*_(*x*_*j*_ − *p*_*ij*_)^2^)	Hartman 3	MN	3	[0, 1]	−3.8628
*f*_20_(*x*)=−∑_*i*=1_^4^(*c*_*i*_exp(−∑_*j*−1_^6^*a*_*ij*_(*x*_*j*_ − *p*_*ij*_)^2^)	Hartman 6	MN	6	[0, 1]	−3.32
*f*_21_(*x*)=−∑_*i*=1_^5^[(*X* − *a*_*i*_)(*X* − *a*_*i*_)^*T*^+*c*_*i*_]^−1^	Langermann 5	MN	4	[0, 10]	−10.1532
*f*_22_(*x*)=−∑_*i*=1_^7^[(*X* − *a*_*i*_)(*X* − *a*_*i*_)^*T*^+*c*_*i*_]^−1^	Langermann 7	MN	4	[0, 10]	−10.4029
*f*_23_(*x*)=−∑_*i*=1_^10^[(*X* − *a*_*i*_)(*X* − *a*_*i*_)^*T*^+*c*_*i*_]^−1^	Langermann 10	MN	4	[0, 10]	−10.5364

**Table 2:** Algorithm parameter setting.

Algorithm	Parameters
MPA	*P*=0.5, FADs=0.2
MFO	*a*=−1 (linearly decreased over iterations)
SSA	*P*=0.2, *C*=0.2
EO	*a*_1_=2, *a*_2_=1
MRFO	*S*=2
HHO	—
GSA	*G*_0_=100, *R*_norm_=2, *R*_power_=1
GWO	*a*=2 (linearly decreased over iterations)

**Table 3:** Ten-chaotic-mapping information.

ID	Mapping type	Function
1	Chebyshev map	*x*_*i*+1_=cos(*i* cos^−1^(*x*_*i*_))
2	Circle map	*x*_*i*+1_=mod(*xi*+*b* − (*a*/2*π*)sin(2*πx*_*k*_), 1), *a*=0.5 and *b*=0.2
3	Gauss map	$x_{i+1}=\left\{\begin{array}{cc} 1 & x_{i}=0\\ 1/\mathrm{mod}\left(x_{i},1\right) & \text{otherwaise} \end{array}\right.$
4	Iterative map	*x*_*i*+1_=sin(*aπ*/*x*_*i*_), *a*=0.7
5	Logistic map	*x*_*i*+1_=*ax*_*i*_(1 − *x*_*i*_), *a*=4
6	Pricewise map	$x_{i+1}=\left\{\begin{array}{cc} x_{i}/p & 0\leq x_{i}<p\\ \left(x_{i}-p\right)/\left(0.5-p\right) & p\leq x_{i}<0.5\\ \left(1-x_{i}-p\right)/\left(0.5-p\right) & 0.5\leq x_{i}<1-p\\ \left(1-x_{i}\right)/\left(p\right) & 1-p\leq x_{i}<1 \end{array}\right.$
7	Sine map	*x*_*i*+1_=*a*/4 · sin(*πx*_*i*_), *a*=4
8	Singer map	*x*_*i*+1_=*μ*(7.86*x*_*i*_ − 23.32*x*_*i*_^2^+28.75*x*_*i*_^3^ − 13.301875*x*_*i*_^4^), *μ*=1.07
9	Sinusoidal map	*x*_*i*+1_=*ax*_*i*_^2^sin(*πx*_*i*_), *a*=2.3
10	Tent map	$x_{i+1}=\left\{\begin{array}{cc} x_{i}/0.7 & x_{i}<0.7\\ 10/3\times \left(1-x_{i}\right) & x_{i}\geq 0.7 \end{array}\right.$

**Table 4:** Results of 10 chaotic maps on all benchmark functions.

*F*(*x*)	Measure	SMA	SMA-C1	SMA-C2	SMA-C3	SMA-C4	SMA-C5	SMA-C6	SMA-C7	SMA-C8	SMA-C9	SMA-C10
F1	Mean	4.25*E* − 317	0.00*E* + 00	0.00*E* + 00	0.00*E* + 00	0.00*E* + 00	0.00*E* + 00	0.00*E* + 00	0.00*E* + 00	0.00*E* + 00	0.00*E* + 00	0.00*E* + 00
	Std	0.00*E* + 00	0.00*E* + 00	0.00*E* + 00	0.00*E* + 00	0.00*E* + 00	0.00*E* + 00	0.00*E* + 00	0.00*E* + 00	0.00*E* + 00	0.00*E* + 00	0.00*E* + 00
F2	Mean	3.31*E* − 139	0.00*E* + 00	1.19*E* − 318	0.00*E* + 00	6.21*E* − 317	1.78*E* − 288	0.00*E* + 00	0.00*E* + 00	2.90*E* − 319	1.60*E* − 311	0.00*E* + 00
	Std	2.34*E* − 138	0.00*E* + 00	0.00*E* + 00	0.00*E* + 00	0.00*E* + 00	0.00*E* + 00	0.00*E* + 00	0.00*E* + 00	0.00*E* + 00	0.00*E* + 00	0.00*E* + 00
F3	Mean	1.58*E* − 322	0.00*E* + 00	0.00*E* + 00	0.00*E* + 00	0.00*E* + 00	0.00*E* + 00	0.00*E* + 00	0.00*E* + 00	0.00*E* + 00	0.00*E* + 00	0.00*E* + 00
	Std	0.00*E* + 00	0.00*E* + 00	0.00*E* + 00	0.00*E* + 00	0.00*E* + 00	0.00*E* + 00	0.00*E* + 00	0.00*E* + 00	0.00*E* + 00	0.00*E* + 00	0.00*E* + 00
F4	Mean	2.40*E* − 140	1.69*E* − 285	3.76*E* − 264	1.59*E* − 281	1.08*E* − 304	5.84*E* − 294	1.25*E* − 312	3.718*E* − 310	5.50*E* − 290	1.50*E* − 272	5.79*E* − 305
	Std	1.70*E* − 139	0.00*E* + 00	0.00*E* + 00	0.00*E* + 00	0.00*E* + 00	0.00*E* + 00	0.00*E* + 00	0.00*E* + 00	0.00*E* + 00	0.00*E* + 00	0.00*E* + 00
F5	Mean	5.97*E* + 00	6.86*E* − 01	6.46*E* − 01	7.08*E* − 01	1.16*E* − 01	6.26*E* − 01	1.19*E* + 00	2.28*E* − 01	1.05*E* − 01	1.19*E* + 00	1.15*E* − 01
	Std	1.04*E* + 01	4.07*E* + 00	3.94*E* + 00	3.73*E* + 00	1.65*E* − 01	3.95*E* + 00	5.40*E* + 00	9.78*E* − 01	1.57*E* − 01	5.19*E* + 00	2.79*E* − 01
F6	Mean	5.39*E* − 03	1.10*E* − 04	1.17*E* − 04	1.13*E* − 04	1.22*E* − 04	1.14*E* − 04	1.06*E* − 04	1.12*E* − 04	1.08*E* − 04	1.13*E* − 04	1.06*E* − 04
	Std	3.23*E* − 03	5.56*E* − 05	5.07*E* − 05	6.74*E* − 05	6.10*E* − 05	5.74*E* − 05	5.23*E* − 05	6.79*E* − 05	4.55*E* − 05	5.95*E* − 05	4.87*E* − 05
F7	Mean	1.99*E* − 04	4.01*E* − 05	2.88*E* − 05	4.32*E* − 05	3.62*E* − 05	3.56*E* − 05	2.57*E* − 05	4.28*E* − 05	3.39*E* − 05	2.78*E* − 05	4.95*E* − 05
	Std	1.75*E* − 04	3.02*E* − 05	2.42*E* − 05	3.80*E* − 05	3.41*E* − 05	2.83*E* − 05	2.68*E* − 05	4.63*E* − 05	3.00*E* − 05	2.78*E* − 05	5.01*E* − 05

F8	Mean	−1.26*E* + 04	−1.26*E* + 04	−1.26*E* + 04	−1.26*E* + 04	−1.26*E* + 04	−1.26*E* + 04	−1.26*E* + 04	−1.26*E* + 04	−1.26*E* + 04	−1.26*E* + 04	−1.26*E* + 04
	Std	4.05*E* − 01	4.05*E* − 02	2.71*E* − 02	2.43*E* − 02	4.94*E* − 02	2.13*E* − 02	3.62*E* − 02	2.41*E* − 02	3.10*E* − 02	2.86*E* − 02	2.15*E* − 02
F9	Mean	0.00*E* + 00	0.00*E* + 00	0.00*E* + 00	0.00*E* + 00	0.00*E* + 00	0.00*E* + 00	0.00*E* + 00	0.00*E* + 00	0.00*E* + 00	0.00*E* + 00	0.00*E* + 00
	Std	0.00*E* + 00	0.00*E* + 00	0.00*E* + 00	0.00*E* + 00	0.00*E* + 00	0.00*E* + 00	0.00*E* + 00	0.00*E* + 00	0.00*E* + 00	0.00*E* + 00	0.00*E* + 00
F10	Mean	8.88*E* − 16	8.88*E* − 16	8.88*E* − 16	8.88*E* − 16	8.88*E* − 16	8.88*E* − 16	8.88*E* − 16	8.88*E* − 16	8.88*E* − 16	8.88*E* − 16	8.88*E* − 16
	Std	0.00*E* + 00	0.00*E* + 00	0.00*E* + 00	0.00*E* + 00	0.00*E* + 00	0.00*E* + 00	0.00*E* + 00	0.00*E* + 00	0.00*E* + 00	0.00*E* + 00	0.00*E* + 00
F11	Mean	0.00*E* + 00	0.00*E* + 00	0.00*E* + 00	0.00*E* + 00	0.00*E* + 00	0.00*E* + 00	0.00*E* + 00	0.00*E* + 00	0.00*E* + 00	0.00*E* + 00	0.00*E* + 00
	Std	0.00*E* + 00	0.00*E* + 00	0.00*E* + 00	0.00*E* + 00	0.00*E* + 00	0.00*E* + 00	0.00*E* + 00	0.00*E* + 00	0.00*E* + 00	0.00*E* + 00	0.00*E* + 00
F12	Mean	3.33*E* − 03	1.48*E* − 05	1.39*E* − 04	1.17*E* − 05	1.08*E* − 05	9.13*E* − 06	1.19*E* − 05	1.78*E* − 04	1.15*E* − 05	1.94*E* − 05	1.54*E* − 05
	Std	5.08*E* − 03	2.24*E* − 05	9.02*E* − 04	6.63*E* − 06	8.30*E* − 06	5.52*E* − 06	1.10*E* − 05	8.02*E* − 04	8.61*E* − 06	4.11*E* − 05	1.60*E* − 05
F13	Mean	7.07*E* − 03	9.56*E* − 04	3.75*E* − 04	1.45*E* − 03	3.80*E* − 04	3.69*E* − 04	1.43*E* − 03	1.60*E* − 04	1.70*E* − 03	1.09*E* − 03	5.73*E* − 04
	Std	1.53*E* − 02	2.86*E* − 03	1.57*E* − 03	4.18*E* − 03	1.59*E* − 03	1.66*E* − 03	4.60*E* − 03	1.19*E* − 04	3.94*E* − 03	3.06*E* − 03	2.18*E* − 03

F14	Mean	9.98*E* − 01	9.98*E* − 01	9.98*E* − 01	9.98*E* − 01	9.98*E* − 01	9.98*E* − 01	9.98*E* − 01	9.98*E* − 01	9.98*E* − 01	9.98*E* − 01	9.98*E* − 01
	Std	1.42*E* − 12	7.21*E* − 14	4.64*E* − 14	9.73*E* − 14	4.15*E* − 14	6.11*E* − 14	1.12*E* − 13	8.66*E* − 14	4.45*E* − 14	6.47*E* − 14	7.89*E* − 14
F15	Mean	5.85*E* − 04	4.35*E* − 04	4.20*E* − 04	4.85*E* − 04	4.58*E* − 04	4.28*E* − 04	4.34*E* − 04	4.61*E* − 04	4.44*E* − 04	4.19*E* − 04	4.31*E* − 04
	Std	2.70*E* − 04	1.56*E* − 04	1.18*E* − 04	2.37*E* − 04	1.90*E* − 04	1.87*E* − 04	1.38*E* − 04	2.21*E* − 04	1.83*E* − 04	1.86*E* − 04	1.69*E* − 04
F16	Mean	−1.03*E* + 00	−1.03*E* + 00	−1.03*E* + 00	−1.03*E* + 00	−1.03*E* + 00	−1.03*E* + 00	−1.03*E* + 00	−1.03*E* + 00	−1.03*E* + 00	−1.03*E* + 00	−1.03*E* + 00
	Std	1.58*E* − 09	1.33*E* − 09	5.15*E* − 11	6.70*E* − 11	8.03*E* − 11	2.64*E* − 10	9.28*E* − 11	4.88*E* − 11	9.90*E* − 11	2.38*E* − 10	5.83*E* − 11
F17	Mean	3.98*E* − 01	3.98*E* − 01	3.98*E* − 01	3.98*E* − 01	3.98*E* − 01	3.98*E* − 01	3.98*E* − 01	3.98*E* − 01	3.98*E* − 01	3.98*E* − 01	3.98*E* − 01
	Std	2.66*E* − 08	2.45*E* − 09	4.31*E* − 09	9.68*E* − 10	2.80*E* − 09	1.25*E* − 09	2.99*E* − 09	3.22*E* − 09	2.14*E* − 09	1.76*E* − 09	1.17*E* − 09
F18	Mean	3.00*E* + 00	3.00*E* + 00	3.00*E* + 00	3.00*E* + 00	3.00*E* + 00	3.00*E* + 00	3.00*E* + 00	3.00*E* + 00	3.00*E* + 00	3.00*E* + 00	3.00*E* + 00
	Std	7.54*E* − 11	5.22*E* − 07	6.07*E* − 07	6.42*E* − 07	3.34*E* − 07	3.80*E* − 07	1.44*E* − 06	2.70*E* − 07	4.03*E* − 07	4.52*E* − 07	7.69*E* − 07
F19	Mean	−3.86*E* + 00	−3.86*E* + 00	−3.86*E* + 00	−3.86*E* + 00	−3.86*E* + 00	−3.86*E* + 00	−3.86*E* + 00	−3.86*E* + 00	−3.86*E* + 00	−3.86*E* + 00	−3.86*E* + 00
	Std	2.77*E* − 07	1.96*E* − 06	1.88*E* − 06	1.71*E* − 06	2.17*E* − 06	9.06*E* − 07	1.29*E* − 06	1.79*E* − 06	3.01*E* − 06	1.77*E* − 06	1.41*E* − 06
F20	Mean	−3.24*E* + 00	−3.26*E* + 00	−3.25*E* + 00	−3.26*E* + 00	−3.26*E* + 00	−3.24*E* + 00	−3.24*E* + 00	−3.25*E* + 00	−3.25*E* + 00	−3.25*E* + 00	−3.23*E* + 00
	Std	5.72*E* − 02	6.10*E* − 02	6.03*E* − 02	6.21*E* − 02	6.10*E* − 02	5.86*E* − 02	5.61*E* − 02	6.02*E* − 02	6.13*E* − 02	5.98*E* − 02	5.49*E* − 02
F21	Mean	−1.02*E* + 01	−1.02*E* + 01	−1.02*E* + 01	−1.02*E* + 01	−1.02*E* + 01	−1.02*E* + 01	−1.02*E* + 01	−1.02*E* + 01	−1.02*E* + 01	−1.02*E* + 01	−1.02*E* + 01
	Std	3.18*E* − 04	7.93*E* − 06	1.19*E* − 05	8.78*E* − 06	1.22*E* − 05	1.77*E* − 05	1.56*E* − 05	9.99*E* − 06	1.19*E* − 05	1.93*E* − 05	8.11*E* − 06
F22	Mean	−1.04*E* + 01	−1.04*E* + 01	−1.04*E* + 01	−1.04*E* + 01	−1.04*E* + 01	−1.04*E* + 01	−1.04*E* + 01	−1.04*E* + 01	−1.04*E* + 01	−1.04*E* + 01	−1.04*E* + 01
	Std	2.97*E* − 04	8.60*E* − 06	1.65*E* − 05	2.86*E* − 05	9.23*E* − 06	9.51*E* − 06	9.87*E* − 06	1.46*E* − 05	6.55*E* − 06	9.76*E* − 06	1.43*E* − 05
F23	Mean	−1.05*E* + 01	−1.05*E* + 01	−1.05*E* + 01	−1.05*E* + 01	−1.05*E* + 01	−1.05*E* + 01	−1.05*E* + 01	−1.05*E* + 01	−1.05*E* + 01	−1.05*E* + 01	−1.05*E* + 01
	Std	3.10*E* − 04	1.08*E* − 05	7.86*E* − 06	1.40*E* − 05	8.20*E* − 06	5.95*E* − 06	8.98*E* − 06	5.22*E* − 06	9.32*E* − 06	6.80*E* − 06	1.50*E* − 05

**Table 5:** Friedman test results for ten chaotic mappings.

*F*(*x*)	SMA-C1	SMA-C2	SMA-C3	SMA-C4	SMA-C5	SMA-C6	SMA-C7	SMA-C8	SMA-C9	SMA-C10	SMA
F1–F7	4.00	5.29	5.00	4.71	5.00	2.29	3.14	3.14	5.29	2.86	11.00
F8–F13	4.17	3.17	2.83	3.00	1.33	4.00	3.17	3.83	4.00	3.00	6.00
F14–F23	5.90	6.40	6.00	4.70	5.00	7.30	5.00	5.80	5.40	5.70	8.80
F1–F23	4.87	5.22	4.87	4.26	4.04	4.91	3.96	4.48	5.00	4.13	8.74

**Table 6:** MSMA variants with different improvement strategies.

Algorithm	COBL	SA	SS
SMA	No	No	No
MSMA-1	Yes	No	No
MSMA-2	No	Yes	No
MSMA-3	No	No	Yes
MSMA	Yes	Yes	Yes

**Table 7:** The statistical results of MSMA-derived algorithms on classical test functions.

*F*(*x*)	Measure	SMA	MSMA-1	MSMA-2	MSMA-3	MSMA
F1	Mean	4.25*E* − 317	0.00*E* + 00	0.00*E* + 00	4.38*E* − 227	0.00*E* + 00
	Std	0.00*E* + 00	0.00*E* + 00	0.00*E* + 00	0.00*E* + 00	0.00*E* + 00
F2	Mean	3.31*E* − 139	0.00*E* + 00	0.00*E* + 00	8.07*E* − 118	8.78*E* − 169
	Std	2.34*E* − 138	0.00*E* + 00	0.00*E* + 00	5.68*E* − 117	0.00*E* + 00
F3	Mean	1.58*E* − 322	0.00*E* + 00	0.00*E* + 00	2.52*E* − 213	0.00*E* + 00
	Std	0.00*E* + 00	0.00*E* + 00	0.00*E* + 00	0.00*E* + 00	0.00*E* + 00
F4	Mean	2.40*E* − 140	4.23*E* − 295	3.84*E* − 277	9.79*E* − 116	1.37*E* − 166
	Std	1.70*E* − 139	0.00*E* + 00	0.00*E* + 00	6.90*E* − 115	0.00*E* + 00
F5	Mean	5.97*E* + 00	7.32*E* − 01	5.00*E* + 00	2.20*E* + 00	3.10*E* − 03
	Std	1.04*E* + 01	4.10*E* + 00	1.04*E* + 01	7.36*E* + 00	8.40*E* − 03
F6	Mean	5.39*E* − 03	1.06*E* − 04	8.33*E* − 03	1.65*E* − 02	2.62*E* − 05
	Std	3.23*E* − 03	5.69*E* − 05	2.52*E* − 02	5.78*E* − 02	1.77*E* − 04
F7	Mean	1.99*E* − 04	4.04*E* − 05	1.60*E* − 04	2.15*E* − 04	4.48*E* − 05
	Std	1.75*E* − 04	3.94*E* − 05	1.90*E* − 04	2.77*E* − 04	4.83*E* − 05

F8	Mean	−1.26*E* + 04	−1.26*E* + 04	−1.26*E* + 04	−1.26*E* + 04	−1.26*E* + 04
	Std	4.05*E* − 01	2.77*E* − 02	1.68*E* − 02	1.44*E* − 03	1.70*E* − 02
F9	Mean	0.00*E* + 00	0.00*E* + 00	0.00*E* + 00	0.00*E* + 00	0.00*E* + 00
	Std	0.00*E* + 00	0.00*E* + 00	0.00*E* + 00	0.00*E* + 00	0.00*E* + 00
F10	Mean	8.88*E* − 16	8.88*E* − 16	8.88*E* − 16	8.88*E* − 16	8.88*E* − 16
	Std	0.00*E* + 00	0.00*E* + 00	0.00*E* + 00	0.00*E* + 00	0.00*E* + 00
F11	Mean	0.00*E* + 00	0.00*E* + 00	0.00*E* + 00	0.00*E* + 00	0.00*E* + 00
	Std	0.00*E* + 00	0.00*E* + 00	0.00*E* + 00	0.00*E* + 00	0.00*E* + 00
F12	Mean	3.33*E* − 03	1.08*E* − 05	5.43*E* − 04	1.20*E* − 04	7.92*E* − 08
	Std	5.08*E* − 03	6.97*E* − 06	1.54*E* − 03	6.35*E* − 04	9.56*E* − 08
F13	Mean	7.07*E* − 03	1.03*E* − 03	2.20*E* − 02	3.05*E* − 03	8.62*E* − 04
	Std	1.53*E* − 02	3.63*E* − 03	9.07*E* − 02	6.13*E* − 03	3.64*E* − 03

F14	Mean	9.98*E* − 01	9.98*E* − 01	9.98*E* − 01	9.98*E* − 01	9.98*E* − 01
	Std	1.42*E* − 12	4.63*E* − 14	5.84*E* − 16	3.28*E* − 16	2.21*E* − 16
F15	Mean	5.85*E* − 04	4.64*E* − 04	5.50*E* − 04	5.54*E* − 04	4.28*E* − 04
	Std	2.70*E* − 04	1.83*E* − 04	2.72*E* − 04	3.76*E* − 04	3.26*E* − 04
F16	Mean	−1.03*E* + 00	−1.03*E* + 00	−1.03*E* + 00	−1.03*E* + 00	− 1.03*E* + 00
	Std	1.58*E* − 09	5.23*E* − 11	1.11*E* − 12	4.60*E* − 16	4.54*E* − 16
F17	Mean	3.98*E* − 01	3.98*E* − 01	3.98*E* − 01	3.98*E* − 01	3.98*E* − 01
	Std	2.66*E* − 08	7.65*E* − 10	1.76*E* − 11	3.36*E* − 16	3.36*E* − 16
F18	Mean	3.00*E* + 00	3.00*E* + 00	3.00*E* + 00	3.00*E* + 00	3.00*E* + 00
	Std	7.54*E* − 11	2.91*E* − 07	8.52*E* − 07	2.52*E* − 14	2.01*E* − 14
F19	Mean	−3.86*E* + 00	−3.86*E* + 00	−3.86*E* + 00	−3.86*E* + 00	− 3.86*E* + 00
	Std	2.77*E* − 07	2.46*E* − 06	6.61*E* − 06	3.26*E* − 09	1.75*E* − 12
F20	Mean	−3.24*E* + 00	−3.25*E* + 00	−3.26*E* + 00	−3.26*E* + 00	− 3.27*E* + 00
	Std	5.72*E* − 02	6.45*E* − 02	6.17*E* − 02	6.12*E* − 02	5.95*E* − 02
F21	Mean	−1.02*E* + 01	−1.02*E* + 01	−1.02*E* + 01	−1.02*E* + 01	−1.02*E* + 01
	Std	3.18*E* − 04	2.08*E* − 05	1.04*E* − 07	3.63*E* − 14	5.13*E* − 14
F22	Mean	−1.04*E* + 01	−1.04*E* + 01	−1.04*E* + 01	−1.04*E* + 01	−1.04*E* + 01
	Std	2.97*E* − 04	1.32*E* − 05	1.43*E* − 07	9.10*E* − 15	1.05*E* − 13
F23	Mean	−1.05*E* + 01	−1.05*E* + 01	−1.05*E* + 01	−1.05*E* + 01	−1.05*E* + 01
	Std	3.10*E* − 04	9.74*E* − 06	7.08*E* − 08	8.74*E* − 15	6.67*E* − 14

**Table 8:** Friedman test results for MSMA-derived algorithms.

*F*(*x*)	SMA	MSMA-1	MSMA-2	MSMA-3	MSMA
F1–F7	4.00	1.29	2.29	4.71	1.71
F8–F13	2.83	1.83	2.50	1.67	1.17
F14–F23	4.60	3.80	3.40	1.60	1.40
F1–F23	3.96	2.52	2.83	2.57	1.43

**Table 9:** Comparison of results on F1–F13 with 30D.

*F*(*x*)	Measure	GWO	GSA	HHO	MRFO	EO	SSA	MFO	MPA	SMA	MSMA
F1	Mean	1.29*E* − 27	2.10*E* − 05	8.36*E* − 96	0.00*E* + 00	7.66*E* − 41	2.06*E* − 07	1.61*E* + 03	1.54*E* − 30	1.29*E* − 288	**0.00*E* + 00**
	Std	1.17*E* − 27	1.49*E* − 04	5.77*E* − 95	0.00*E* + 00	2.81*E* − 40	2.97*E* − 07	3.70*E* + 03	9.64*E* − 30	0.00*E* + 00	0.00*E* + 00
F2	Mean	9.44*E* − 17	1.51*E* − 01	3.66*E* − 49	2.96*E* − 207	5.93*E* − 24	2.31*E* + 00	3.59*E* + 01	9.52*E* − 18	5.27*E* − 147	2.89*E* − 164
	Std	7.62*E* − 17	4.92*E* − 01	2.52*E* − 48	0.00*E* + 00	5.77*E* − 24	1.64*E* + 00	2.24*E* + 01	2.27*E* − 17	3.73*E* − 146	0.00*E* + 00
F3	Mean	1.19*E* − 05	1.03*E* + 03	5.16*E* − 77	0.00*E* + 00	5.04*E* − 09	1.44*E* + 03	1.91*E* + 04	1.74*E* − 16	1.43*E* − 293	**0.00*E* + 00**
	Std	2.55*E* − 05	3.61*E* + 02	2.49*E* − 76	0.00*E* + 00	1.45*E* − 08	7.65*E* + 02	1.24*E* + 04	8.29*E* − 16	0.00*E* + 00	0.00*E* + 00
F4	Mean	6.03*E* − 07	6.94*E* + 00	1.68*E* − 48	4.53*E* − 199	4.75*E* − 10	1.26*E* + 01	6.96*E* + 01	5.88*E* − 16	1.32*E* − 143	6.72*E* − 161
	Std	7.38*E* − 07	1.98*E* + 00	8.39*E* − 48	0.00*E* + 00	1.34*E* − 09	4.24*E* + 00	9.70*E* + 00	1.82*E* − 15	9.33*E* − 143	4.75*E* − 160
F5	Mean	2.70*E* + 01	5.78*E* + 01	9.11*E* − 03	2.27*E* + 01	2.54*E* + 01	3.10*E* + 02	4.81*E* + 06	2.55*E* + 01	9.10*E* + 00	2.56*E* − 02
	Std	7.05*E* − 01	6.24*E* + 01	1.25*E* − 02	5.56*E* − 01	1.98*E* − 01	4.22*E* + 02	1.92*E* + 07	5.57*E* − 01	1.16*E* + 01	1.31*E* − 01
F6	Mean	8.33*E* − 01	2.42*E* − 16	1.62*E* − 04	7.89*E* − 11	6.81*E* − 06	2.73*E* − 07	2.82*E* + 03	1.54*E* − 03	5.00*E* − 03	7.93*E* − 07
	Std	4.26*E* − 01	1.47*E* − 16	1.72*E* − 04	1.99*E* − 10	4.88*E* − 06	6.06*E* − 07	6.09*E* + 03	1.09*E* − 02	3.01*E* − 03	1.70*E* − 06
F7	Mean	2.22*E* − 03	8.92*E* − 02	1.16*E* − 04	1.28*E* − 04	1.48*E* − 03	1.77*E* − 01	3.50*E* + 00	1.40*E* − 03	1.88*E* − 04	**4.79*E* − 05**
	Std	1.36*E* − 03	4.46*E* − 02	9.09*E* − 05	1.02*E* − 04	8.31*E* − 04	7.02*E* − 02	5.92*E* + 00	8.75*E* − 04	1.79*E* − 04	4.17*E* − 05

F8	Mean	−5.96*E* + 03	−2.69*E* + 03	−1.24*E* + 04	−8.17*E* + 03	−8.96*E* + 03	−7.43*E* + 03	−8.66*E* + 03	−9.09*E* + 03	−1.26*E* + 04	−**1.26*E* + 04**
	Std	8.24*E* + 02	5.09*E* + 02	9.08*E* + 02	7.49*E* + 02	5.89*E* + 02	6.96*E* + 02	8.90*E* + 02	4.94*E* + 02	3.77*E* − 01	1.77*E* − 02
F9	Mean	2.90*E* + 00	2.93*E* + 01	0.00*E* + 00	0.00*E* + 00	0.00*E* + 00	5.24*E* + 01	1.62*E* + 02	0.00*E* + 00	0.00*E* + 00	**0.00*E* + 00**
	Std	4.52*E* + 00	7.30*E* + 00	0.00*E* + 00	0.00*E* + 00	0.00*E* + 00	1.78*E* + 01	3.66*E* + 01	0.00*E* + 00	0.00*E* + 00	0.00*E* + 00
F10	Mean	1.03*E* − 13	1.19*E* − 08	8.88*E* − 16	8.88*E* − 16	8.63*E* − 15	2.53*E* + 00	1.56*E* + 01	1.53*E* − 15	8.88*E* − 16	**8.88*E* − 16**
	Std	1.69*E* − 14	2.71*E* − 09	0.00*E* + 00	0.00*E* + 00	2.23*E* − 15	6.39*E* − 01	6.60*E* + 00	3.18*E* − 15	0.00*E* + 00	0.00*E* + 00
F11	Mean	5.42*E* − 03	2.82*E* + 01	0.00*E* + 00	0.00*E* + 00	0.00*E* + 00	1.58*E* − 02	2.08*E* + 01	0.00*E* + 00	0.00*E* + 00	**0.00*E* + 00**
	Std	1.05*E* − 02	7.27*E* + 00	0.00*E* + 00	0.00*E* + 00	0.00*E* + 00	1.15*E* − 02	3.76*E* + 01	0.00*E* + 00	0.00*E* + 00	0.00*E* + 00
F12	Mean	4.47*E* − 02	1.92*E* + 00	1.21*E* − 05	2.80*E* − 12	6.27*E* − 07	7.79*E* + 00	4.32*E* + 01	8.04*E* − 05	4.75*E* − 03	7.58*E* − 08
	Std	2.49*E* − 02	1.17*E* + 00	2.22*E* − 05	4.49*E* − 12	5.39*E* − 07	3.23*E* + 00	1.66*E* + 02	3.07*E* − 04	7.41*E* − 03	1.10*E* − 07
F13	Mean	6.57*E* − 01	9.94*E* + 00	1.52*E* − 04	2.25*E* + 00	1.90*E* − 02	1.49*E* + 01	8.20*E* + 06	4.90*E* − 02	7.96*E* − 03	8.61*E* − 04
	Std	2.35*E* − 01	7.66*E* + 00	3.62*E* − 04	1.23*E* + 00	3.19*E* − 02	1.43*E* + 01	5.80*E* + 07	6.74*E* − 02	1.22*E* − 02	3.63*E* − 03

**Table 10:** Comparison of results on F1–F13 with 100D.

*F*(*x*)	Measure	GWO	GSA	HHO	MRFO	EO	SSA	MFO	MPA	SMA	MSMA
F1	Mean	1.33*E* − 12	4.13*E* + 03	3.86*E* − 90	0.00*E* + 00	4.47*E* − 29	1.45*E* + 03	6.16*E* + 04	1.16*E* − 28	1.74*E* − 246	0.00*E* + 00
	Std	1.12*E* − 12	9.39*E* + 02	2.73*E* − 89	0.00*E* + 00	6.92*E* − 29	5.34*E* + 02	1.52*E* + 04	3.55*E* − 28	0.00*E* + 00	0.00*E* + 00
F2	Mean	4.01*E* − 08	1.73*E* + 01	6.85*E* − 50	1.76*E* − 200	1.58*E* − 17	4.61*E* + 01	2.41*E* + 02	8.70*E* − 16	1.80*E* − 124	2.39*E* − 200
	Std	1.59*E* − 08	3.70*E* + 00	2.41*E* − 49	0.00*E* + 00	1.41*E* − 17	8.01*E* + 00	3.81*E* + 01	1.78*E* − 15	1.27*E* − 123	0.00*E* + 00
F3	Mean	5.43*E* + 02	1.48*E* + 04	2.22*E* − 58	0.00*E* + 00	8.10*E* + 00	5.44*E* + 04	2.33*E* + 05	1.35*E* − 13	1.82*E* − 276	0.00*E* + 00
	Std	6.01*E* + 02	3.60*E* + 03	1.52*E* − 57	0.00*E* + 00	1.88*E* + 01	2.45*E* + 04	5.10*E* + 04	4.56*E* − 13	0.00*E* + 00	0.00*E* + 00
F4	Mean	1.01*E* + 00	1.81*E* + 01	2.84*E* − 48	1.82*E* − 198	5.57*E* − 02	2.83*E* + 01	9.30*E* + 01	2.32*E* − 14	1.54*E* − 134	1.18*E* − 146
	Std	1.33*E* + 00	2.10*E* + 00	1.74*E* − 47	0.00*E* + 00	3.76*E* − 01	3.63*E* + 00	2.33*E* + 00	6.16*E* − 14	1.09*E* − 133	8.34*E* − 146
F5	Mean	9.78*E* + 01	1.01*E* + 05	4.49*E* − 02	9.46*E* + 01	9.66*E* + 01	1.64*E* + 05	1.67*E* + 08	9.71*E* + 01	4.07*E* + 01	2.37*E* + 00
	Std	6.73*E* − 01	5.38*E* + 04	5.40*E* − 02	9.85*E* − 01	1.06*E* + 00	9.49*E* + 04	6.46*E* + 07	7.43*E* − 01	4.00*E* + 01	1.37*E* + 01
F6	Mean	1.01*E* + 01	3.99*E* + 03	5.07*E* − 04	8.61*E* − 01	3.78*E* + 00	1.37*E* + 03	5.98*E* + 04	3.79 *E* + 00	1.47*E* + 00	1.57*E* − 02
	Std	9.48*E* − 01	8.20*E* + 02	6.39*E* − 04	4.38*E* − 01	5.64*E* − 01	3.49*E* + 02	1.30*E* + 04	8.39*E* − 01	1.54*E* + 00	4.79*E* − 02
F7	Mean	6.57*E* − 03	3.66*E* + 00	1.79*E* − 04	1.45*E* − 04	2.49*E* − 03	2.73*E* + 00	2.70*E* + 02	1.71*E* − 03	3.13*E* − 04	4.68*E* − 05
	Std	3.04*E* − 03	1.70*E* + 00	2.00*E* − 04	1.23*E* − 04	1.21*E* − 03	6.03*E* − 01	1.15*E* + 02	9.14*E* − 04	2.84*E* − 04	4.60*E* − 05

F8	Mean	−1.65*E* + 04	−4.59*E* + 03	−4.19*E* + 04	−2.37*E* + 04	−2.59*E* + 04	−2.11*E* + 04	−2.35*E* + 04	−2.53*E* + 04	−4.19*E* + 04	−4.19*E* + 04
	Std	2.78*E* + 03	6.46*E* + 02	4.13*E* + 00	1.53*E* + 03	1.49*E* + 03	1.89*E* + 03	2.09*E* + 03	1.10*E* + 03	1.62*E* + 01	3.52*E* + 00
F9	Mean	1.03*E* + 01	1.72*E* + 02	0.00*E* + 00	0.00*E* + 00	2.27*E* − 15	2.45*E* + 02	8.68*E* + 02	0.00*E* + 00	0.00*E* + 00	0.00*E* + 00
	Std	7.34*E* + 00	1.79*E* + 01	0.00*E* + 00	0.00*E* + 00	1.61*E* − 14	4.22*E* + 01	7.20*E* + 01	0.00*E* + 00	0.00*E* + 00	0.00*E* + 00
F10	Mean	1.30*E* − 07	4.43*E* + 00	8.88*E* − 16	8.88*E* − 16	3.41*E* − 14	1.01*E* + 01	1.99*E* + 01	3.09*E* − 15	8.88*E* − 16	8.88*E* − 16
	Std	5.07*E* − 08	6.21*E* − 01	0.00*E* + 00	0.00*E* + 00	5.55*E* − 15	1.35*E* + 00	1.29*E* − 01	2.37*E* − 15	0.00*E* + 00	0.00*E* + 00
F11	Mean	1.44*E* − 03	6.86*E* + 02	0.00*E* + 00	0.00*E* + 00	2.54*E* − 04	1.44*E* + 01	5.66*E* + 02	0.00*E* + 00	0.00*E* + 00	0.00*E* + 00
	Std	5.78*E* − 03	4.40*E* + 01	0.00*E* + 00	0.00*E* + 00	1.80*E* − 03	3.69*E* + 00	1.20*E* + 02	0.00*E* + 00	0.00*E* + 00	0.00*E* + 00
F12	Mean	2.91*E* − 01	1.00*E* + 01	3.73*E* − 06	5.30*E* − 03	3.93*E* − 02	3.33*E* + 01	3.10*E* + 08	4.82*E* − 02	1.03*E* − 02	9.55*E* − 06
	Std	7.04*E* − 02	3.73*E* + 00	5.96*E* − 06	2.65*E* − 03	9.47*E* − 03	1.25*E* + 01	1.79*E* + 08	1.10*E* − 02	1.69*E* − 02	1.63*E* − 05
F13	Mean	6.66*E* + 00	2.06*E* + 03	1.81*E* − 04	9.87*E* + 00	5.80*E* + 00	9.59*E* + 03	6.38*E* + 08	8.92*E* + 00	1.73*E* − 01	1.22*E* − 03
	Std	4.21*E* − 01	4.00*E* + 03	2.26*E* − 04	1.22*E* − 01	1.02*E* + 00	1.91*E* + 04	3.28*E* + 08	5.38*E* − 01	4.24*E* − 01	3.59*E* − 03

**Table 11:** Comparison of results on F1–F13 with 500D.

*F*(*x*)	Measure	GWO	GSA	HHO	MRFO	EO	SSA	MFO	MPA	SMA	MSMA
F1	Mean	1.70*E* − 03	5.31*E* + 04	2.41*E* − 90	0.00*E* + 00	1.05*E* − 22	9.42*E* + 04	1.16*E* + 06	3.40*E* − 25	3.86*E* − 229	0.00*E* + 00
	Std	4.97*E* − 04	4.22*E* + 03	1.71*E* − 89	0.00*E* + 00	1.31*E* − 22	6.70*E* + 03	3.39*E* + 04	1.32*E* − 24	0.00*E* + 00	0.00*E* + 00
F2	Mean	1.09*E* − 02	7.96*E* + 266	3.58*E* − 48	5.55*E* − 200	7.18*E* − 14	5.36*E* + 02	1.49*E* + 125	1.55*E* + 01	6.53*E* − 01	7.97*E* − 02
	Std	1.54*E* − 03	1.35*E* + 116	1.33*E* − 47	0.00*E* + 00	4.44*E* − 14	2.08*E* + 01	1.05*E* + 126	5.01*E* + 01	2.37*E* + 00	4.94*E* − 01
F3	Mean	3.42*E* + 05	1.32*E* + 06	5.24*E* − 33	0.00*E* + 00	2.93*E* + 04	1.42*E* + 06	4.90*E* + 06	3.76*E* − 08	4.11*E* − 166	0.00*E* + 00
	Std	9.35*E* + 04	7.64*E* + 05	3.71*E* − 32	0.00*E* + 00	3.71*E* + 04	6.85*E* + 05	8.91*E* + 05	2.60*E* − 07	0.00*E* + 00	0.00*E* + 00
F4	Mean	6.58*E* + 01	2.77*E* + 01	1.14*E* − 46	3.29*E* − 195	7.66*E* + 01	4.04*E* + 01	9.89*E* + 01	5.35*E* − 13	4.25*E* − 95	1.66*E* − 166
	Std	5.95*E* + 00	1.74*E* + 00	7.58*E* − 46	0.00*E* + 00	1.67*E* + 01	2.87*E* + 00	4.13*E* − 01	1.09*E* − 12	3.00*E* − 94	0.00*E* + 00
F5	Mean	4.98*E* + 02	7.35*E* + 06	2.03*E* − 01	4.96*E* + 02	4.97*E* + 02	3.80*E* + 07	5.06*E* + 09	4.97*E* + 02	2.23*E* + 02	7.01*E* + 00
	Std	3.14*E* − 01	1.18*E* + 06	1.99*E* − 01	6.47*E* − 01	3.86*E* − 01	4.87*E* + 06	2.25*E* + 08	2.60*E* − 01	2.09*E* + 02	1.40*E* + 01
F6	Mean	9.11*E* + 01	5.28*E* + 04	1.79*E* − 03	6.38*E* + 01	8.70*E* + 01	9.37*E* + 04	1.15*E* + 06	7.48*E* + 01	2.45*E* + 01	3.02*E* − 01
	Std	2.13*E* + 00	3.62*E* + 03	2.56*E* − 03	2.71*E* + 00	1.71*E* + 00	7.19*E* + 03	4.05*E* + 04	1.96*E* + 00	2.99*E* + 01	5.49*E* − 01
F7	Mean	4.85*E* − 02	8.33*E* + 02	1.39*E* − 04	1.12*E* − 04	4.56*E* − 03	2.72*E* + 02	3.85*E* + 04	1.80*E* − 03	5.40*E* − 04	4.90*E* − 05
	Std	1.15*E* − 02	1.34*E* + 02	1.18*E* − 04	9.88*E* − 05	1.83*E* − 03	4.42*E* + 01	2.06*E* + 03	1.18*E* − 03	4.84*E* − 04	3.96*E* − 05

F8	Mean	−5.48*E* + 04	−1.06*E* + 04	−2.09*E* + 05	−7.44*E* + 04	−7.57*E* + 04	−6.03*E* + 04	−6.15*E* + 04	−8.44*E* + 04	−2.09*E* + 05	−2.09*E* + 05
	Std	1.14*E* + 04	1.87*E* + 03	1.13*E* + 02	3.87*E* + 03	5.60*E* + 03	6.06*E* + 03	4.79*E* + 03	3.49*E* + 03	2.52*E* + 02	1.25*E* + 02
F9	Mean	6.90*E* + 01	2.66*E* + 03	0.00*E* + 00	0.00*E* + 00	5.46*E* − 14	3.17*E* + 03	6.99*E* + 03	0.00*E* + 00	0.00*E* + 00	0.00*E* + 00
	Std	2.05*E* + 01	1.29*E* + 02	0.00*E* + 00	0.00*E* + 00	2.18*E* − 13	1.22*E* + 02	1.17*E* + 02	0.00*E* + 00	0.00*E* + 00	0.00*E* + 00
F10	Mean	1.83*E* − 03	1.02*E* + 01	8.88*E* − 16	8.88*E* − 16	5.26*E* − 13	1.42*E* + 01	2.03*E* + 01	1.69*E* − 14	8.88*E* − 16	8.88*E* − 16
	Std	3.85*E* − 04	2.57*E* − 01	0.00*E* + 00	0.00*E* + 00	2.53*E* − 13	2.30*E* − 01	1.45*E* − 01	2.01*E* − 14	0.00*E* + 00	0.00*E* + 00
F11	Mean	1.21*E* − 02	8.62*E* + 03	0.00*E* + 00	0.00*E* + 00	9.77*E* − 17	8.51*E* + 02	1.05*E* + 04	0.00*E* + 00	0.00*E* + 00	0.00*E* + 00
	Std	3.27*E* − 02	1.90*E* + 02	0.00*E* + 00	0.00*E* + 00	3.64*E* − 17	5.07*E* + 01	2.84*E* + 02	0.00*E* + 00	0.00*E* + 00	0.00*E* + 00
F12	Mean	7.46*E* − 01	9.10*E* + 03	1.80*E* − 06	2.86*E* − 01	5.83*E* − 01	1.36*E* + 06	1.19*E* + 10	3.95*E* − 01	1.28*E* − 02	3.40*E* − 05
	Std	4.60*E* − 02	1.25*E* + 04	2.69*E* − 06	2.81*E* − 02	2.70*E* − 02	6.59*E* + 05	7.05*E* + 08	2.86*E* − 02	2.83*E* − 02	6.32*E* − 05
F13	Mean	5.08*E* + 01	2.89*E* + 06	5.03*E* − 04	4.97*E* + 01	4.92*E* + 01	3.64*E* + 07	2.22*E* + 10	4.85*E* + 01	1.62*E* + 00	2.49*E* − 02
	Std	1.63*E* + 00	1.02*E* + 06	1.03*E* − 03	1.28*E* − 02	2.92*E* − 01	8.94*E* + 06	1.32*E* + 09	2.65*E* − 01	4.92*E* + 00	6.70*E* − 02

**Table 12:** Comparison of results on F1–F13 with 1000D.

*F*(*x*)	Measure	GWO	GSA	HHO	MRFO	EO	SSA	MFO	MPA	SMA	MSMA
F1	Mean	6.10*E* − 02	4.74*E* + 03	1.22*E* − 93	0.00*E* + 00	7.49*E* − 21	1.03*E* + 04	4.67*E* + 04	6.72*E* − 25	0.00*E* + 00	0.00*E* + 00
	Std	7.28*E* − 01	1.46*E* + 295	3.02*E* − 48	2.16*E* − 199	4.37*E* − 13	1.19*E* + 03	1.34*E* + 03	1.16*E* + 03	8.28*E* − 01	0.00*E* + 00
F2	Mean	4.62*E* − 01	3.12*E* + 01	1.97*E* − 47	0.00*E* + 00	2.26*E* − 13	2.77*E* + 01	3.70*E* + 02	1.49*E* + 02	3.00*E* + 00	1.50*E* − 01
	Std	1.52*E* + 06	7.43*E* + 06	3.46*E* − 28	0.00*E* + 00	1.49*E* + 05	5.41*E* + 06	1.79*E* + 07	2.88*E* − 09	2.69*E* − 74	5.21*E* − 01
F3	Mean	2.88*E* + 05	4.35*E* + 06	2.44*E* − 27	0.00*E* + 00	1.14*E* + 05	2.88*E* + 06	3.39*E* + 06	1.49*E* − 08	1.90*E* − 73	0.00*E* + 00
	Std	7.76*E* + 01	3.29*E* + 01	7.44*E* − 49	1.34*E* − 192	8.19*E* + 01	4.40*E* + 01	9.95*E* + 01	1.90*E* − 12	1.60*E* − 69	0.00*E* + 00
F4	Mean	3.54*E* + 00	1.54*E* + 00	4.14*E* − 48	0.00*E* + 00	1.34*E* + 01	2.16*E* + 00	1.91*E* − 01	3.78*E* − 12	1.13*E* − 68	2.65*E* − 159
	Std	1.05*E* + 03	2.09*E* + 07	4.21*E* − 01	9.96*E* + 02	9.97*E* + 02	1.17*E* + 08	1.25*E* + 10	9.97*E* + 02	5.41*E* + 02	1.86*E* − 158
F5	Mean	2.85*E* + 01	2.10*E* + 06	5.40*E* − 01	3.97*E* − 01	9.14*E* − 02	1.10*E* + 07	3.23*E* + 08	1.70*E* − 01	4.05*E* + 02	2.46*E* + 01
	Std	2.03*E* + 02	1.24*E* + 05	7.29*E* − 03	1.71*E* + 02	2.06*E* + 02	2.35*E* + 05	2.72*E* + 06	1.84*E* + 02	4.44*E* + 01	4.94*E* + 01
F6	Mean	2.93*E* + 00	4.93*E* + 03	1.10*E* − 02	4.29*E* + 00	1.85*E* + 00	1.02*E* + 04	5.03*E* + 04	3.31*E* + 00	6.32*E* + 01	1.48*E* + 00
	Std	1.57*E* − 01	5.57*E* + 03	1.80*E* − 04	1.56*E* − 04	5.42*E* − 03	1.73*E* + 03	1.97*E* + 05	1.83*E* − 03	6.49*E* − 04	2.84*E* + 00
F7	Mean	3.28*E* − 02	4.96*E* + 02	1.74*E* − 04	1.32*E* − 04	2.79*E* − 03	1.94*E* + 02	5.88*E* + 03	9.24*E* − 04	5.76*E* − 04	4.72*E* − 05
	Std	−8.59*E* + 04	−1.43*E* + 04	−4.19*E* + 05	−1.08*E* + 05	−1.14*E* + 05	−9.00*E* + 04	−8.84*E* + 04	−1.31*E* + 05	−4.19*E* + 05	3.96*E* − 05

F8	Mean	1.43*E* + 04	2.81*E* + 03	3.16*E* + 01	6.45*E* + 03	6.62*E* + 03	8.66*E* + 03	6.69*E* + 03	4.36*E* + 03	3.88*E* + 02	−4.19*E* + 05
	Std	1.96*E* + 02	6.64*E* + 03	0.00*E* + 00	0.00*E* + 00	2.18*E* − 13	7.64*E* + 03	1.54*E* + 04	0.00*E* + 00	0.00*E* + 00	1.66*E* + 02
F9	Mean	4.95*E* + 01	2.03*E* + 02	0.00*E* + 00	0.00*E* + 00	5.97*E* − 13	1.70*E* + 02	2.14*E* + 02	0.00*E* + 00	0.00*E* + 00	0.00*E* + 00
	Std	1.77*E* − 02	1.10*E* + 01	8.88*E* − 16	8.88*E* − 16	2.12*E* − 12	1.45*E* + 01	2.04*E* + 01	3.88*E* − 14	8.88*E* − 16	0.00*E* + 00
F10	Mean	2.67*E* − 03	1.62*E* − 01	0.00*E* + 00	0.00*E* + 00	1.13*E* − 12	1.74*E* − 01	2.06*E* − 01	3.40*E* − 14	0.00*E* + 00	8.88*E* − 16
	Std	4.99*E* − 02	2.05*E* + 04	0.00*E* + 00	0.00*E* + 00	1.58*E* − 16	2.14*E* + 03	2.45*E* + 04	0.00*E* + 00	0.00*E* + 00	0.00*E* + 00
F11	Mean	7.67*E* − 02	2.65*E* + 02	0.00*E* + 00	0.00*E* + 00	5.54*E* − 17	8.74*E* + 01	4.61*E* + 02	0.00*E* + 00	0.00*E* + 00	0.00*E* + 00
	Std	1.24*E* + 00	1.23*E* + 05	1.63*E* − 06	5.39*E* − 01	8.10*E* − 01	1.09*E* + 07	3.06*E* + 10	6.16*E* − 01	5.61*E* − 03	0.00*E* + 00
F12	Mean	2.71*E* − 01	8.72*E* + 04	2.53*E* − 06	3.34*E* − 02	1.76*E* − 02	3.80*E* + 06	1.10*E* + 09	2.37*E* − 02	1.15*E* − 02	1.79*E* − 05
	Std	5.04*E* + 01	3.00*E* + 06	4.45*E* − 04	4.97*E* + 01	4.91*E* + 01	3.58*E* + 07	2.24*E* + 10	4.85*E* + 01	1.31*E* + 00	3.38*E* − 05
F13	Mean	1.52*E* + 00	1.15*E* + 06	6.53*E* − 04	2.92*E* − 02	2.73*E* − 01	7.45*E* + 06	1.02*E* + 09	2.94*E* − 01	2.38*E* + 00	2.17*E* − 02
	Std	1.63*E* + 00	1.02*E* + 06	1.03*E* − 03	1.28*E* − 02	2.92*E* − 01	8.94*E* + 06	1.32*E* + 09	2.65*E* − 01	4.92*E* + 00	5.43*E* − 02

**Table 13:** Comparison of results on F14–F23 with fixed dimension.

*F*(*x*)	Measure	GWO	GSA	HHO	MRFO	EO	SSA	MFO	MPA	SMA	MSMA
F14	Mean	4.43*E* + 00	5.16*E* + 00	1.27*E* + 00	9.98*E* − 01	9.98*E* − 01	1.30*E* + 00	2.52*E* + 00	9.98*E* − 01	9.98*E* − 01	9.98E − 01
	Std	4.15*E* + 00	3.11*E* + 00	1.04*E* + 00	1.05*E* − 16	1.79*E* − 16	6.10*E* − 01	2.03*E* + 00	1.57*E* − 16	6.40*E* − 13	3.45*E* − 16
F15	Mean	2.85*E* − 03	4.64*E* − 03	3.73*E* − 04	3.83*E* − 04	2.39*E* − 03	2.42*E* − 03	1.21*E* − 03	3.07*E* − 04	5.97*E* − 04	3.54*E* − 04
	Std	6.54*E* − 03	2.85*E* − 03	1.35*E* − 04	2.51*E* − 04	6.06*E* − 03	5.37*E* − 03	1.10*E* − 03	5.03*E* − 15	3.14*E* − 04	1.85*E* − 04
F16	Mean	−1.03*E* + 00	−1.03*E* + 00	−1.03*E* + 00	−1.03*E* + 00	−1.03*E* + 00	−1.03*E* + 00	−1.03*E* + 00	−1.03*E* + 00	−1.03*E* + 00	−1.03E + 00
	Std	2.25*E* − 08	4.31*E* − 16	4.15*E* − 09	2.50*E* − 16	3.14*E* − 16	1.75*E* − 14	2.24*E* − 16	6.22*E* − 16	1.23*E* − 09	4.51*E* − 16
F17	Mean	3.98*E* − 01	3.98*E* − 01	3.98*E* − 01	3.98*E* − 01	3.98*E* − 01	3.98*E* − 01	3.98*E* − 01	3.98*E* − 01	3.98*E* − 01	3.98E − 01
	Std	9.38*E* − 07	3.36*E* − 16	7.42*E* − 06	3.36*E* − 16	3.36*E* − 16	9.04*E* − 15	3.36*E* − 16	3.36*E* − 16	3.17*E* − 08	3.36*E* − 16
F18	Mean	3.00*E* + 00	3.00*E* + 00	3.00 *E* + 00	3.00*E* + 00	3.00*E* + 00	3.00*E* + 00	3.00*E* + 00	3.00*E* + 00	3.00*E* + 00	3.00*E* + 00
	Std	3.52*E* − 05	4.61*E* − 15	3.63*E* − 07	1.13*E* − 15	1.37*E* − 15	2.61*E* − 13	1.57*E* − 15	2.12*E* − 15	1.36*E* − 10	2.49*E* − 14
F19	Mean	−3.86*E* + 00	−3.86*E* + 00	−3.86*E* + 00	−3.86*E* + 00	−3.86*E* + 00	−3.86*E* + 00	−3.86*E* + 00	−3.86*E* + 00	−3.86*E* + 00	−3.86E + 00
	Std	2.67*E* − 03	2.69*E* − 15	2.65*E* − 03	3.12*E* − 15	2.90*E* − 15	7.50*E* − 11	3.14*E* − 15	2.78*E* − 15	2.47*E* − 07	3.29*E* − 12
F20	Mean	−3.26*E* + 00	−3.32*E* + 00	−3.07*E* + 00	−3.26*E* + 00	−3.26*E* + 00	−3.23*E* + 00	−3.22*E* + 00	−3.32*E* + 00	−3.25*E* + 00	−3.26*E* + 00
	Std	7.69*E* − 02	3.11*E* − 16	1.27*E* − 01	6.00*E* − 02	6.58*E* − 02	5.79*E* − 02	6.39*E* − 02	1.10*E* − 11	5.92*E* − 02	6.05*E* − 02
F21	Mean	−9.24*E* + 00	−6.44*E* + 00	−5.23*E* + 00	−8.42*E* + 00	−8.94*E* + 00	−7.44*E* + 00	−6.39*E* + 00	−1.02*E* + 01	−1.02*E* + 01	−1.02E + 01
	Std	2.14*E* + 00	3.63*E* + 00	9.15*E* − 01	2.44*E* + 00	2.50*E* + 00	3.20*E* + 00	3.36*E* + 00	4.01*E* − 11	2.08*E* − 04	1.08*E* − 13
F22	Mean	−1.02*E* + 01	−1.01*E* + 01	−5.12*E* + 00	−8.78*E* + 00	−8.59*E* + 00	−9.18*E* + 00	−7.49*E* + 00	−1.04*E* + 01	−1.04*E* + 01	−1.04E + 01
	Std	1.05*E* + 00	1.42*E* + 00	8.74*E* − 01	2.51*E* + 00	3.01*E* + 00	2.67*E* + 00	3.39*E* + 00	2.83*E* − 11	2.88*E* − 04	5.61*E* − 14
F23	Mean	−1.04*E* + 01	−1.02*E* + 01	−5.07*E* + 00	−9.13*E* + 00	−9.73*E* + 00	−7.13*E* + 00	−7.74*E* + 00	−1.05*E* + 01	−1.05*E* + 01	−1.05E + 01
	Std	1.15*E* + 00	1.56*E* + 00	3.83*E* − 01	2.40*E* + 00	2.22*E* + 00	3.67*E* + 00	3.51*E* + 00	4.75*E* − 11	3.33*E* − 04	7.30*E* − 14

**Table 14:** Statistical results of Wilcoxon's rank-sum test.

MSMA VS.		F1–F13 (Dim = 30)	F1–F13 (Dim = 100)	F1–F13 (Dim = 500)	F1–F13 (Dim = 1000)	F14–F23
Wilcoxon's rank-sum test (+/=/−)	GWO	12/1/0	13/0/0	13/0/0	13/0/0	8/2/0
	GSA	12/0/1	13/0/0	13/0/0	13/0/0	6/2/2
	HHO	10/3/0	5/5/3	4/5/4	4/5/4	9/1/1
	MRFO	6/7/0	7/6/0	6/6/1	6/6/1	6/2/2
	EO	11/2/0	11/2/0	11/2/0	11/2/0	6/3/1
	SSA	12/0/1	13/0/0	13/0/0	13/0/0	10/0/0
	MFO	13/0/0	13/0/0	13/0/0	13/0/0	6/2/2
	MPA	8/4/1	11/2/0	11/2/0	11/2/0	6/3/1
	SMA	7/6/0	10/3/0	10/3/0	9/4/0	10/0/0
	Sum	91/23/3	96/18/3	94/18/5	93/19/5	66/15/9

**Table 15:** Statistical results of the Friedman test.

			GWO	GSA	HHO	MRFO	EO	SSA	MFO	MPA	SMA	MSMA
F1–F13	Dim = 30	Friedman value	7.23	7.85	3.27	2.96	4.85	8.31	9.62	5.08	3.65	2.19
		Friedman rank	7	8	3	2	5	9	10	6	4	1
	Dim = 100	Friedman value	7.00	8.54	2.58	2.96	5.31	8.62	9.69	5.15	3.19	1.96
		Friedman rank	7	8	2	3	6	9	10	5	4	1
	Dim = 500	Friedman value	7.00	8.31	2.35	3.04	5.85	8.54	9.69	4.69	3.27	2.27
		Friedman rank	8	8	2	3	6	9	10	5	4	1
	Dim = 1000	Friedman value	7.00	8.31	2.35	3.04	5.92	8.38	9.85	4.69	3.27	2.19
		Friedman rank	8	9	2	3	6	9	10	5	4	1

F14–F23	Fixed dim.	Friedman value	7.30	5.40	8.70	4.05	4.70	7.30	6.05	3.00	5.80	2.70
		Friedman rank	8	5	10	3	4	8	7	2	6	1

F1–F23	All dim.	Friedman value	7.24	7.52	3.99	3.11	4.83	8.17	9.14	4.76	3.94	2.30
		Friedman rank	7	8	4	2	6	9	10	5	3	1

**Table 16:** Comparison results of the MSMA for the welded beam design problem.

Algorithm	Optimal values for variables				Optimal cost
	*x*_1_	*x*_2_	*x*_3_	*x*_4_	
DDSCA [51]	0.20516	3.4759	9.0797	0.20552	1.7305
HGA [52]	0.205712	3.470391	9.039693	0.205716	1.725236
MGWO-III [53]	0.205667	3.471899	9.036679	0.205733	1.724984
IAPSO [54]	0.205729	3.470886	9.036623	0.205729	**1.724852**
TEO [55]	0.205681	3.472305	9.035133	0.205796	1.725284
hHHO-SCA [56]	0.190086	3.696496	9.386343	0.204157	1.779032
HPSO [57]	0.20573	3.470489	9.036624	0.20573	**1.724852**
CPSO [58]	0.202369	3.544214	9.048210	0.205723	1.728024
WCA [59]	0.205728	3.470522	9.036620	0.205729	1.724856
SaDN [60]	0.2444	6.21787	8.2915	0.2444	1.9773
MSMA	0.205729	3.470488	9.036623	0.205729	**1.724852**

**Table 17:** Comparison results of the MSMA for the tension/compression spring design problem.

Algorithm	Optimal values for variables			Optimal cost
	*x*_1_	*x*_2_	*x*_3_	
GA3 [61]	0.051989	0.363965	10.890522	0.0126810
SaDN [60]	0.051622	0.355105	11.384415	0.012665
CPSO [58]	0.051728	0.357644	11.244543	0.0126747
CDE [62]	0.051609	0.354714	11.410831	0.0126702
DDSCA [51]	0.052669	0.380673	10.0153	0.012688
GSA [24]	0.050276	0.323680	13.525410	0.0127022
hHHO-SCA [56]	0.054693	0.433378	7.891402	0.0128229
AEO [63]	0.051897	0.361751	10.879842	0.012667
MVO [64]	0.05251	0.3762	10.33513	0.012970
MSMA	0.051808	0.35959	11.210570	0.012665

## Data Availability

The data used to support the findings of this study are included within the article.
